# Sea surface currents and geographic isolation shape the genetic population structure of a coral reef fish in the Indian Ocean

**DOI:** 10.1371/journal.pone.0193825

**Published:** 2018-03-09

**Authors:** Filip Huyghe, Marc Kochzius

**Affiliations:** Marine Biology, Ecology and Biodiversity, Vrije Universiteit Brussel (VUB), Brussels, Belgium; University of California Santa Cruz, UNITED STATES

## Abstract

In this contribution, we determine the genetic population structure in the Skunk Clownfish (*Amphiprion akallopsisos*) across the Indian Ocean, and on a smaller geographic scale in the Western Indian Ocean (WIO). Highly restricted gene flow was discovered between populations on either side of the Indian Ocean using the control region as a mitochondrial marker (mtDNA). We verify this conclusion using 13 microsatellite markers and infer fine scale genetic structuring within the WIO. In total 387 samples from 21 sites were analysed using mtDNA and 13 microsatellite loci. Analysis included estimation of genetic diversity and population differentiation. A haplotype network was inferred using mtDNA. Nuclear markers were used in Bayesian clustering and a principal component analysis. Both markers confirmed strong genetic differentiation between WIO and Eastern Indian Ocean (EIO) populations, and a shallower population structure among Malagasy and East African mainland populations. Limited gene flow across the Mozambique Channel may be explained by its complex oceanography, which could cause local retention of larvae, limiting dispersal between Madagascar and the East African coast. Two other potential current-mediated barriers to larval dispersal suggested in the WIO, the split of the SEC at approximately 10° S and the convergence of the Somali Current with the East African Coast Current at approximately 3° S, were not found to form a barrier to gene flow in this species.

## Introduction

Coral reefs are threatened worldwide by several human induced factors, such as overfishing, tourism, or pollution. They are at risk to convert to an alternative, macroalgae-dominated state that would alter their ecosystem characteristics. This process is further accelerated by human-induced environmental stresses, such as global warming and ocean acidification, making urgent conservation measures a necessity [1]. A very efficient and much used conservation instrument is the establishment of marine protected areas (MPAs), protecting certain coral reef zones from fishing pressure and other potentially harmful activities. Correctly established and managed MPAs serve several purposes: they fulfil a role as sanctuaries for both species and genetic diversity, but are also intended to strengthen ecosystem resilience and promote recovery after disturbances beyond their boundaries. To fulfil this dual role, the spacing of MPAs should take into account the trade-off between the protection of sites that are not connected by gene flow and have a unique genetic composition, and well-connected sites that promote ecosystem resilience better [2]. In addition, MPA spacing should respect the balance between promoting resilience through well connected MPAs and providing spill-over of new recruits to exploited adjacent areas and benefit fisheries [3].

Since gene flow is almost exclusively realised through larval dispersal in coral reef associated organisms, patterns of larval dispersal have been studied to evaluate connectivity in these organisms using various methods, such as population genetics and phylogeography, direct tagging of larvae, parentage analysis, otolith chemistry, and biophysical models [4, 5]. An overall pattern of larval dispersal has not been described yet, and empirical data obtained so far describe different degrees and configurations of population structure, indicating that larval dispersal may be influenced by a multitude of still unknown environmental and behavioural factors. Consequently, conservation managers are urged to plan MPA spacing in a precautious manner and scientists to continue gathering information of larval dispersal and connectivity among marine populations [6].

**Fig 1:**
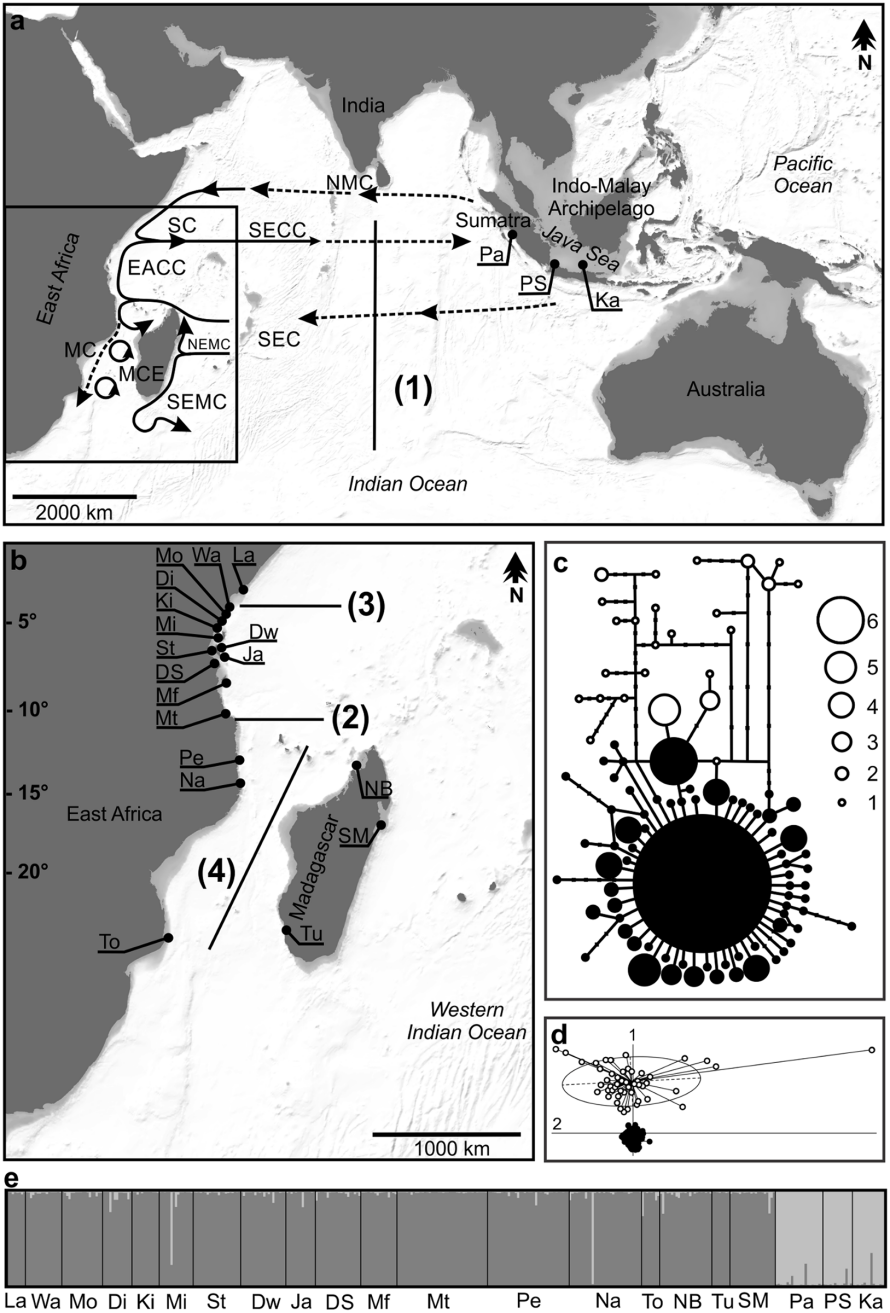
Sampling sites, haplotype network, PCA and structure bar plot. **(A)** Map of the Indian Ocean and (B) Western Indian Ocean (WIO) showing the sample sites for *Amphiprion akallopisos* and prevailing currents during the Northeast Monsoon: EACC: East African Coast Current; MC: Mozambique Current; MCE: Mozambique Channel Eddies; NEMC: Northeast Madagascar Current; NMC: Northeast Monsoon Current; SC: Somali Current; SECC: South Equatorial Counter Current [13, 14]. Potential distance- or current-mediated barriers to larval dispersal indicated with black lines (1): large area of open ocean separating the EIO from the WIO; (2): split of the SEC at approximately 10° S; (3) Confluence of EACC and SC; (4) Mozambique Channel Eddies. Sampling site codes as in Table 1. (C) Haplotype network based on control region sequences. Black circles represent haplotypes from in the WIO and white circles from the EIO; size of the circles corresponds to the number of individuals as indicated, with the largest circle representing 217 individuals; lines represent one, small dots additional mutational steps. (D) Scatterplot of the Principal Component Analysis (PCA) based on microsatellite data; black circles represent individuals from in the WIO and white circles from the EIO;1 (vertical): first axis; 2 (horizontal): second axis. (E) Bar plot showing each individual’s estimated membership fraction to each of two clusters (K = 2) and the distribution of these clusters among sampling sites (codes as in Table 1) as revealed in Bayesian clustering implemented in STRUCTURE.

Results of population genetic studies of reef associated organisms spanning the whole Indian Ocean basin display a wide variety of population structure, with some revealing the existence of a barrier to gene flow between the Western Indian Ocean (WIO) and the Eastern Indian Ocean (EIO) [7–10], while others detect highly connected populations on both sides of the Indian Ocean [11, 12]. Because of the importance of the pelagic larval phase for dispersal, patterns are likely to be influenced by sea surface currents, moving drifting larvae in a specific direction. In the WIO, the prevailing South Equatorial Current (SEC) flows from East to West across the Indian Ocean until it reaches Madagascar, where one component splits into the North (NEMC) and South-East Madagascar Current (SEMC), while another one is joint by the NEMC at the Northern tip of Madagascar (Fig 1). This component of the SEC continues West until it reaches the East African Coast at approximately ten degrees South, close to the border between Tanzania and Mozambique. There, it splits into the southward Mozambique Current (MC), which creates a number of eddies on its way through the Mozambique Channel, and the northward East African Coast Current (EACC), which converges with the seasonal (November-April) southward Somali Current (SC) at the northern coast of Kenya, joining into the South Equatorial Counter Current (SECC) that flows to the East (Fig 1) [13, 14]. This current regime has the potential to create barriers to gene flow in three different regions of the WIO: First, at 10° S between reefs North and South, where the SEC splits into the MC and EACC. Limited gene flow across this barrier has been demonstrated, albeit the actual zone of differentiation was located more to the South, around Central Mozambique, in one study [15], and more to the North in another [10]. Second, at the northern coast of Kenya, where the EACC and SC meet and create an off-shore current flowing East. Limited gene flow across this second potential barrier to dispersal was found in two different reef fish species [16, 17]. Third, the westward direction of the SEC and the eddies in the Mozambique Channel could form a barrier to dispersal among populations along the East African Coast and populations at the various islands in the Indian Ocean. Indications of limited gene flow across this barrier have been encountered repeatedly in reef associated and coastal species [17, 18]. However, several other studies on coral reef associated species have revealed high connectivity and gene flow across all of these barriers, leading to meta-populations lacking genetic structure across the WIO [19–22].

Another factor capable of influencing the results of a phylogeographic study is the choice of genetic markers. Because of its matrilineal inheritance, mtDNA is likely to be more severely influenced by bottleneck events in populations compared to nuclear DNA due to lower effective population size. Consequently, mtDNA diversity is often very low in populations having suffered bottlenecks and may not have returned to equilibrium state yet after such an event. This will mask more recent genetic differentiation and means that mtDNA will often only provide evidence for historical gene flow [23]. The use of mtDNA in phylogeographic studies was further criticised, because the effect of bottlenecks on genetic diversity in populations of different animal species was found to be very variable, independent from species abundance. This must be seen as an indication that mitochondrial genetic markers are under selection pressure in many animal species [24]. Although it was later added that this phenomenon was stronger in certain taxa than in others [25], there is a risk that the neutrality assumption is not respected for phylogeographic studies based on mitochondrial markers. However, lower effective population size in mtDNA makes the effect of genetic drift stronger on this locus, which contributes to a shorter coalescence time [26]. For example, in a fish species with a large population size and a recent colonisation history like *Pleuronectes platessa*, a mtDNA marker enabled to define population structure more precisely than nuclear markers, i.e. microsatellite loci [27]. Microsatellite markers were criticised, because their high mutation rate, repetitive nature, and complex mutation process increase the risk of homoplasy and cause other interpretative problems, such as null alleles, lowered F_st_-values and allele drop out [26, 28]. Microsatellite loci, however, are abundant in most genomes, making it possible to conduct multi-locus studies, and thus making analyses more robust against coalescent stochasticity and selective sweeps, for which single locus mitochondrial markers are more vulnerable [29]. Considering advantages and disadvantages of both types of markers, a combination of both can be seen as the ideal strategy to infer population genetic structure in most cases [30].

In studies where both marker types are simultaneously used, mito-nuclear discordance, or differences between results from mtDNA and nuclear markers, is common. Several explanations, such as introgression, sex-biased dispersal, selection, and different effective population sizes have been suggested [31]. More specifically, an analysis of 14 studies on reef fishes revealed that only in four data sets mtDNA and microsatellite markers were congruent. In six studies, microsatellites revealed a population structure while mtDNA did not, and the opposite occurred in four other studies [32]. In the WIO, microsatellite markers revealed population structure in the soldierfish *Myripristis berndtii*, while mtDNA did not [17], but in the snapper *Lutjanus kasmira* both markers were congruent [22]. A few studies using both marker types have been conducted on clownfish. In a study on *A*. *ocellaris* in the Indo-Malay Archipelago, results from both markers were congruent, but population structure inferred from mtDNA was more pronounced [33]. In three other studies, however, the opposite was true, with microsatellite results revealing genetic differentiation not detected using mtDNA [34–36].

The population structure of the skunk clownfish has been studied using mtDNA, showing highly restricted gene flow between populations in the WIO and the EIO. However, only a weak genetic population structure was detected in the WIO and none in the EIO [9]. In this study, we complement this analysis using thirteen microsatellite loci. Furthermore, the existing WIO dataset was extended with samples from three different sites in Mozambique and one additional site in Madagascar in order to span all three potential current-mediated barriers to gene flow in this region. We aim to investigate whether: (1) the genetic break between WIO and EIO is also detected using microsatellite markers; (2) barriers to gene flow could be detected within the WIO using this extended dataset and a combination of different markers.

## Materials and methods

### Sampling and DNA extraction

A total of 387 *A*. *akallopisos* individuals were sampled between one and 25 m depth at 18 sites in the WIO and three in the EIO (Table 1 and Fig 1a and 1b). Fish were caught with two hand nets in their host anemone while SCUBA-diving.

**Table 1:** Diversity of mitochondrial control region sequences and microsatellites in *Amphiprion akallopisos* from the Indian Ocean.

Sample site	Country	Code	Control Region					Microsatellites		
			N_ind_	N_h_	N_h_/N_ind_	H	Π (%)	N_ind_	H_o_	Ar
WIO										
Lamu	Kenya	La	8	3	0.375	0.607	0.21	8	0.743	6.00
Watamu	Kenya	Wa	16	7	0.438	0.625	0.34	16	0.771	9.62
Mombasa	Kenya	Mo	18	4	0.222	0.399	0.13	18	0.650	7.92
Diani	Kenya	Di	13	3	0.231	0.295	0.09	13	0.524	5.77
Kisite	Kenya	Ki	12	6	0.500	0.758	0.36	12	0.587	4.92
Misali	Tanzania	Mi	15	7	0.467	0.657	0.36	15	0.611	5.08
Stone Town	Tanzania	St	21	6	0.286	0.495	0.20	21	0.561	5.15
Dongwe	Tanzania	Dw	18	5	0.278	0.484	0.16	20	0.628	6.23
Jambiani	Tanzania	Ja	11	4	0.364	0.491	0.17	13	0.558	4.69
Dar Es Salaam	Tanzania	DS	19	2	0.105	0.199	0.06	20	0.542	5.62
Mafia Island	Tanzania	Mf	15	4	0.267	0.371	0.16	16	0.520	5.69
Mtwara	Tanzania	Mt	37	13	0.351	0.703	0.30	40	0.607	7.23
Pemba	Mozambique	Pe	35	18	0.514	0.770	0.64	36	0.616	7.23
Nacala	Mozambique	Na	31	10	0.323	0.503	0.27	32	0.566	7.23
Tofo	Mozambique	To	8	2	0.250	0.250	0.08	8	0.683	5.15
Nosy Bé	Madagascar	NB	23	7	0.304	0.577	0.26	23	0.668	6.77
Tuléar	Madagascar	Tu	18	7	0.389	0.569	0.30	20	0.647	6.69
Sainte Marie	Madagascar	SM	8	5	0.625	0.786	1.06	8	0.637	4.77
EIO										
Padang	Indonesia	Pa	12	12	1.000	1.000	3.29	21	0.769	11.15
Pulau Seribu	Indonesia	PS	12	11	0.917	0.985	2.64	14	0.680	9.31
Karimun Java	Indonesia	Ka	10	8	0.800	0.956	2.90	13	0.646	8.85

Sample sites, site codes, number of individuals per site (N_ind_), number of haplotypes per site (N_h_), number of haplotypes per number of individuals ratio (N_h_/N_ind_), haplotype diversity(H), nucleotide diversity (Π), observed heterozygosity(H_o_), and allelic richness(Ar).

A small piece of the caudal fin, from 5 mm by 5 mm up to 20 mm by 20 mm, depending on the size of the individual, was removed after which the fish was returned into its host anemone. Samples were preserved in 96% ethanol immediately after the dive. Research permits were obtained from the following institutions: TANZANIA COMMISSION FOR SCIENCE AND TECHNOLOGY (COSTECH) Ali Hassan Mwinyi Road, Kijitonyama Area. P.O. Box 4302, Dar es Salaam, Tanzania; Zanzibar Research Committee, PO Box 239, Stone Town, Zanzibar, Tanzania; Kenya: National Council for Science and Technology, Nairobi, Research permit No: NCST/RRI/12/1/BS/250; Mozambique: Universidade Eduardo Mondlane, Escola de Ciências Marinhas e Costeiras (ECMC), Quelimane; Indonesia: Universitas Hasanuddin, Makassar, Research permit No: 55/PSTK/UH/XI/04; Madagascar: Ministère de l'Environnement, de l'Ecologie et des Forêts; Autorisation de recherche: N°287/15/MEEMF/SG/DGF/DAPT/SCBT. DNA from the samples from Kenya, Tanzania, Indonesia, and Madagascar (partly) was extracted with the QIAGEN (Düsseldorf, Germany) extraction kit, and DNA from samples from Madagascar (Sainte Marie) and Mozambique was extracted with the E.Z.N.A. Tissue DNA Kit (Omega Bio-tek, Norcross, Georgia, USA), following the manufacturer’s protocol.

### Control region (CR)

A fragment of the CR was amplified by polymerase chain reaction (PCR) using the primers CR-A (5’-TTCCACCTCTAACTCCCAAAGCTAG-3’) and CR-E (5’-CCTGAAGTAGGAACCAGATG-3’) [37]. The PCR was conducted in an Eppendorf Ep S Mastercycler with a volume of 50 μl for each PCR that contained 2 μL DNA template, 10 mM Tris-HCl (pH 9), 50 mM KCl, 4 mM MgCl_2_, 0.4 μM of each primer, 0.2 mM dNTPs, and 1 U Taq polymerase. The following temperature profile was used: 94 °C for 5 minutes, followed by 35–40 cycles of 1 minute at 94 °C, 1.5 minutes at 45 °C and 1 min at 72 °C. Final extension was conducted at 72 °C for 5 minutes. Sequencing was done with an ABI 3770XL automated sequencer (Applied Biosystems, Foster City, USA). The sequences were edited using the software ChromasPro (v. 1.5, Technelysium Ltd, UK). A multiple alignment was done using Clustal W [38] as implemented in the software BioEdit v. 7.0.0.1 [39]. Haplotype and nucleotide diversity, F-statistics, as well as analysis of molecular variance (AMOVA) were calculated with the software Arlequin v. 3.5 [40]. Multiple testing can lead to the occurrence of false positives as a consequence of the multitude of tests. We corrected for this multiplicity problem using the False Discovery Rate method that sets a lower significance threshold than p<0.05 in function of the number of independent tests [41]. The program MIGRATE v. 3.11.6 [42] was used to test migration rates in either direction among populations. After initial testing, the following start parameters were chosen to do Bayesian inference with constant mutation rate (since there was only one locus) and Metropolis algorithm to generate posterior distribution. The prior distribution parameters were set at minimum = 0; mean = 500; maximum = 1.000. One long chain was run, with samples taken every 20 steps, and 100.000 samples taken per chain, with a burn-in of 50.000 samples. Five replicates were run per analysis. Four parallel chains were run under a static heating scheme at following start temperatures: 1–1.5–3–100.000. Tests for selective neutrality of the marker, Tajima’s D-test [43] and Fu’s F_s_-test [44], as well as the sum of squared deviation and Harpending’s raggedness index [45] to test Rogers’ model of sudden population expansion [46] were also conducted with the software Arlequin. A haplotype network was constructed using the programme TCS v. 1.21 [47].

### Microsatellites

Primer candidates were identified in a literature study searching for microsatellite loci previously described for the genus *Amphiprion* (S1 Table). All found primer candidates were tested in a subset of the samples for the presence of the same locus in *A*. *akallopisos*. The 13 best amplifying primers were selected and divided into one set of six and one set of seven primer pairs based on the observed length of the PCR product (Table 2).

**Table 2:** Characterization of the 13 microsatellite loci in *Amphiprion akallopisos* with their respective motive, fluorescent label, PCR product length, number of alleles (Na), observed (H_o_) and expected (H_e_) heterozygosities. The first six primers were used in one multiplex set, the next seven in another.

Locus	Primer sequence	Repeat motif	Fluorescent label	Length (bp)	Na	H_o_	H_e_
Am1	F: ACAAAGCCTTCATGTGGGTC	TG	6FAM	90–100	6	0.423	0.321
	R: CGCAAGTGTTGCCTCATAGA						
Am9	F-TGCTGCACTCTGTCTATTTTGT	TTA	6FAM	123–165	14	0.634	0.578
	R-GTGACTGAAGGCAAGGCAAT						
D114	F: TGTTCCAGCTCTGATATTTGAC	GATA	6FAM	192–260	17	0.700	0.691
	R: TTGGCAGTGTTTTATACCTGTC						
120	F-TCGATGACATAACACGACGCAGT	GT	6FAM	452–460	5	0.261	0.279
	R-GACGGCCTCGATCTGCAAGCTGA						
Am17	F-GGCTGTCTGGGATGAGATGT	AATA	VIC	96–178	17	0.696	0.692
	R-TGTTCTGCAGATGGACTGTTTT						
CF11	F: GCTGGTTACAACACCTTG	CT/CA	NED	187–219	17	0.698	0.691
	R: GTAATTGCTGCAAGACAG						
B6	F: TGTCTTCTCCCAAGTCAG	CATC	6FAM	124–168	10	0.614	0.639
	R: ACGAGGCTCAACATACCTG						
61	F- TGAACACATAAACGCTCACTCAC	GT	6FAM	270–372	41	0.739	0.791
	R: AAGACAATGCCTCCACATATCTA						
10TCTA	F: GGGACGTATCTGTTGGAAATGAT	TCTA	6FAM	494–578	22	0.859	0.850
	R: TTAAGGTACTGTGAGATGAGACT						
Am7	F-TGTCGCTACGACAGACTGCT	ATG	VIC	74–86	5	0.460	0.409
	R-GCATGAGTGATTGGACCCTA						
A130	F-GCACTCAACACAAAGACCTTA	CA	VIC	260–322	29	0.610	0.714
	R-ACCCAAACAACATCCAGTC						
44	F-TTGGAGCAGCGTACTTAGCT	GT	NED	224–270	19	0.637	0.634
	R-AGATGTGTTTACGCACGCTT						
D103	F: GTTGGCTAATGGTGCTGTG	GATA	PET	246–306	16	0.890	0.855
	R: GATTCTGTGGTGGCATCAG						

Multiplex PCR was conducted using the four different fluorescent labels 6-FAM, VIC, PET and NED (Applied Biosystems, Foster City, CA, USA) with these two sets of primer pairs. The PCR was conducted in an Eppendorf Ep S Mastercycler with a volume of 12.5 μL for each PCR that contained 2.5 μL DNA template and 10 μL of a master mix containing 1.25 μL primer mix (containing 2 μM of each primer), 6.25 μL Multiplex PCR Master Mix (QIAGEN, Hilden, Germany), containing optimised concentrations of HotStarTaq^®^ Plus DNA Polymerase, MgCl_2_, and dNTPs and Multiplex PCR Plus Buffer (with Factor MP), and 2.5 μL H_2_O. The following temperature profile was used: 95 °C for 5 minutes once, then 95 °C for 30 seconds, 1.5 minutes at 57 °C for annealing and 30 seconds at 72 °C for extension, repeated during 35 cycles. Finally, a last extension was done at 68 °C for 30 minutes. The PCR product was diluted 20 times and mixed with buffer (HiDi) and GeneScan^™^-500 LIZ^®^ size standard (Applied Biosystems, Darmstadt, Germany) and then analysed on an ABI 3100 Automated Sequencer (Applied Biosystems). The resulting electropherograms were scored manually with GeneMarker V2.6.3 (SoftGenetics, State College, PA, USA). The dataset was tested for the presence of null alleles with Microchecker v1.0 [48]. To test for Hardy-Weinberg Equilibrium (HWE), assess genetic diversity, observed and unbiased expected heterozygosity, as well as the number of private alleles per population, we used the program GenAlEx 6.5 [49]. Arlequin was used for F-statistics and to calculate AMOVA. The program MIGRATE v. 3.11.6 [42] was used to test migration rates in either direction among populations. After initial testing, Bayesian inference with constant mutation rate among loci and Metropolis algorithm to generate posterior distribution was run. The prior distribution parameters were set at minimum = 0; mean = 800; maximum = 8.000. One long chain was run, with samples taken every 100 steps, and 500.000 samples taken per chain, with a burn-in of 100.000 samples. Two replicates were run per analysis. Four parallel chains were run under a static heating scheme at following start temperatures: 1–1.5–3–100.000. The software STRUCTURE 2.3.4 [50], which uses a Bayesian clustering based on Markov chain Monte Carlo (MCMC) assignment method, was run without prior population information and under the admixture model to determine the number of genetic clusters (K). STRUCTURE was run for K = 1–21 for the whole dataset, for K = 1–18 for the WIO, and for K = 1–3 for the EIO, using 10 runs with a burn-in length of 100,000 and 1,000,000 MCMC replications. The most likely true number of clusters was determined with Evanno’s test using ΔK [51].

Finally, a Principal Component Analysis (PCA) and Discriminant Analysis of Principal Components (DAPC), which uses an algorithm that clusters individuals in groups optimising between group variance and minimising within-group variance, was done using the program AdeGenet in R [52].

### Assessment of the influence of sampling size and confidence interval on genetic structure

When analysing population genetic structure, the accuracy of the analysis critically depends on the capacity of the sampled individuals to represent the gene pool of the population they originated from [53]. Especially with potentially highly polymorphic markers like microsatellites, there is a risk of overrepresentation of rare alleles, alleles occurring less than 1% in the real population, when the number of sampled individuals per population is low. Typically, when less than 20 individuals are sampled in a population, there is an increased risk that the allele frequencies used to infer population genetic structure do not reflect the real allele frequencies of the population [53]. This in turn can lead to artificially high F_st_-values (or equivalent) and therefore false positive errors in population structure inference, although a higher number of loci seems to temper this effect [53, 54]. Our research involved sampling of natural populations of clownfish in sometimes remote areas. For three of the analysed populations (Lamu, Tofo, and Sainte-Marie), we were not able to analyse more than eight individuals, and for several others an ideal sampling size of 20 individuals could not be reached. The possible influence of reduced sample size on our results was therefore tested with reduced datasets of eight individuals per population, the lowest number of individuals per population encountered. We ran two tests. First, we withheld the first eight individuals of each population and discarded the others. Second, we randomly selected eight individuals per population, using a prize draw site (https://www.dcode.fr/tirage-au-sort-nombre-aleatoire, last accessed 26/12/2017) and discarded the other individuals. We then compared the ϕ_st_, ϕ_ct_, F_st_, and F_ct_-values obtained in these reduced datasets with the values obtained using the complete dataset. We calculated the difference between the value of the complete dataset and the reduced datasets and then tested whether the set of differences was statistically different from 0, using the z-test function in Excel.

Furthermore, it has been pointed out that low ϕ/F_st_-values (< 0.1), even though significantly different from 0, can be misleading as estimates for gene flow [55]. To compensate for this ambiguity, we calculated the 95% confidence intervals of all pairwise ϕ/F_st_-values and F_ct_-values of AMOVA significantly different from 0 in Arlequin using 20.000 permutations and only accepted the ϕ/F_st_-values where the 95% confidence interval did not overlap with 0. Only when the 95% confidence interval does not overlap with 0, it can be accepted as an indication of limited gene flow among populations [56].

## Results

### Control region: Amplification and neutrality testing

Sequences from 74 individuals from three different sites in Mozambique as well as from 23 additional individuals from two different sites in Madagascar were successfully edited and aligned with the 263 sequences from Huyghe & Kochzius (2016) [9], resulting in an alignment of 360 sequences with a length of 337 base pairs. Sequence information was uploaded to the European Nucleotide Archive (accession numbers L824024-824092). Deviation from Hardy-Weinberg equilibrium in both the WIO and the EIO was detected in this enlarged dataset, confirming the results obtained in Huyghe & Kochzius (2016) (Fu’s F & Tajima’s D). For both regions, a recent population expansion was identified with the sum of squared deviation test and Harpending’s raggedness index, again confirming the results of Huyghe & Kochzius (2016).

### Microsatellites: Characterisation and neutrality testing

One of the 16 initially selected primer pairs (AC137; S1 Table) did not produce a PCR product in several individuals and was discarded from further analysis. Of the resulting 15 loci, two (AM10 and AM6; S1 Table) showed signs of null alleles in all populations, and were therefore also removed from the dataset. For the 13 remaining loci (Table 2), there was no indication of linkage disequilibrium and there was no indication for departure from HWE for any of the populations across loci. The number of alleles per locus for the 13 markers used in the analysis varied between 5 (locus 120 & Am7) and 41 (locus 61). Observed heterozygosity ranged between 0.261 (locus 120) and 0.890 (D103) and was very close to expected heterozygosity for all markers. No significant heterozygosity deficit or excess was detected for any of the loci. These results allow us to conclude that the dataset is appropriate for the proposed analysis.

### Genetic diversity: Control region and microsatellites

In the dataset of 360 individuals, 98 haplotypes were identified, of which none occurred simultaneously in the WIO and the EIO (Fig 1c). Just like in Huyghe & Kochzius (2016), however, the WIO and EIO individuals did not form monophyletic groups. Haplotype diversity was much higher in the EIO (1.000 to 0.985) than in the WIO (0.786 to 0.199), as was the haplotype per individual ratio per population (EIO: 1.000–0.800; WIO: 0.625–0.105). Nucleotide diversity was on average an order of magnitude higher and at least more than double as high in the EIO populations (2.64–3.29%) compared to the WIO populations (0.06–1.06%). Within the WIO, the Dar es Salaam population consistently registered the lowest diversity values, and the Sainte Marie population the highest (Table 1). The higher diversity in the EIO can also be seen in the higher divergence, i.e. more mutational steps, among haplotypes found in EIO individuals than in WIO individuals (Fig 1c). In the WIO, 217 out of 312 individuals (69%) belonged to the same dominant haplotype and most other individuals belonged to haplotypes that differed by only one mutational step from this central haplotype, giving the section of the network to which most African individuals belong a typical star like appearance.

As for the microsatellite data, mean observed heterozygosity over all loci was moderate in both the EIO populations (0.769–0.646) and the WIO populations (0.743–0.520). Allelic richness was higher in the EIO (11.15–8.85) than in the WIO (9.62–4.69).

### Genetic structure

#### Control region

The threshold to accept ϕ_st_-values as significantly different from 0 applying the False Discovery Rate approach was set at p<0.0241. All values significantly different from 0 were tested for overlap of the 95% confidence interval with 0 (S2 Table). Only the values without overlap were accepted. The results from Huyghe & Kochzius (2016) regarding population structure in the Indian Ocean were confirmed with an AMOVA (overall ϕ_st_ = 0.24; p < 0.001) and pairwise ϕ_st_-values (Table 3). Strongly restricted gene flow between EIO and WIO populations, already suggested by the absence of shared haplotypes, was detected in this enlarged dataset. Within the WIO, significant population structure was detected by AMOVA (overall ϕ_st_ = 0.029; p < 0.001). Population structure was clearly caused by the differentiation of the population of Sainte Marie at the East coast of Madagascar from the other WIO populations (all pairwise ϕ_st_-values significantly different from 0, except with Lamu, Misali, Jambiani, and Tofo (Table 3)). Hierarchical AMOVA, testing for population structure across potential oceanographic barriers, indicated population structure between EIO and WIO (Barrier 1; Table 4). Within the WIO, there was no indication of population structure across the other three potential oceanographic barriers when explicitly tested (Table 4).

**Table 3:** Pairwise F_st_ (Microsatellites: above diagonal)/ϕ_st_(mtDNA: under diagonal) of *Amphiprion akallopisos* populations from the Indian Ocean with adapted significance levels.

	La	Wa	Mo	Di	Ki	Mi	St	Dw	Ja	DS	Mf
La		-0.001	0.011	0.017	0.022	0.015	0.013	0.007	0.025	0.022	0.026
Wa	0.021		0.031	0.038***	0.049***	0.020	0.029**	0.031***	0.040***	0.044***	0.059***
Mo	0.092	0.012		-0.004	0.012	0.011	-0.005	0.001	0.021	-0.001	0.014
Di	0.092	-0.021	-0.009		0.006	0.019	-0.007	-0.008	0.009	-0.008	0.001
Ki	0.078	0.050	0.096*	0.079		0.006	0.007	0.008	0.015	0.021	0.000
Mi	-0.030	-0.007	0.014	-0.006	0.040		0.002	0.002	0.018	0.009	0.033
St	0.059	0.010	0.009	-0.001	0.065	0.011		-0.009	0.007	-0.010	0.015*
Dw	0.075	-0.007	0.014	0.009	0.087***	0.003	0.009		-0.001	-0.003	0.009
Ja	0.060	-0.010	-0.003	0.004	0.038	-0.022	-0.018	-0.007		0.012	-0.006
DS	0.152	0.017*	0.039	0.029	0.124***	0.020	0.021	0.034	0.039		0.015
Mf	0.065	-0.002	0.015	-0.003	0.026	0.000	-0.005	0.012	-0.011	0.023	
Mt	0.034	0.009	-0.000	-0.010	0.064	0.006	0.016	0.012	-0.018	0.012	0.005
Pe	-0.006	0.004	-0.003	-0.012	0.021	0.001	0.008	0.006	-0.016	0.005	-0.008
Na	0.028	-0.005	-0.009	-0.019	0.060	0.005	-0.000	-0.006	-0.013	-0.002	-0.008
To	0.071	-0.035	-0.079	-0.005	0.045	-0.034	-0.021	-0.009	-0.013	0.035	-0.020
NB	0.043	0.005	-0.011	-0.002	0.072***	-0.005	0.016	0.004	0.000	0.019	0.006
Tu	0.026	0.000	0.009	-0.011	0.056	0.001	0.008	0.008	-0.010	0.012	-0.003
SM	0.164	0.191***	0.271***	0.235***	0.188***	0.182	0.262***	0.258***	0.196	0.310**	0.234**
Pa	0.321***	0.391***	0.425***	0.394***	0.355***	0.389***	0.455***	0.429***	0.355***	0.461***	0.405***
PS	0.336***	0.393***	0.435***	0.408***	0.366***	0.397***	0.468***	0.435***	0.369***	0.475***	0.421***
Ka	0.276***	0.342***	0.384***	0.355***	0.313***	0.342***	0.420***	0.391***	0.318***	0.428***	0.369***
	Mt	Pe	Na	To	NB	Tu	SM	Pa	Ps	Ka	
La	0.008	0.021	0.012	-0.010	0.014	0.024	0.017	0.077***	0.101***	0.112***	
Wa	0.022**	0.026***	0.031***	0.021	0.037***	0.049***	0.028	0.080***	0.108***	0.113***	
Mo	0.005	0.003	-0.002	0.004	0.020*	0.026	0.024	0.098***	0.118***	0.126***	
Di	0.005	0.004	0.000	-0.007	0.031	0.032	0.031	0.098***	0.124***	0.124***	
Ki	0.008	0.011	0.004	0.003	0.031***	0.041***	0.028	0.122***	0.143***	0.151***	
Mi	-0.001	-0.004	0.004	0.007	0.018*	0.021	0.006	0.116***	0.141***	0.147***	
St	-0.003	-0.006	-0.006	-0.013	0.019**	0.014	0.045*	0.097***	0.122***	0.122***	
Dw	-0.007	-0.003	-0.003	-0.019	0.009	0.018*	0.018	0.096***	0.115***	0.117***	
Ja	0.016	0.018	0.009	-0.007	0.015	0.017	0.026	0.101***	0.120***	0.118***	
DS	0.008	0.004	-0.000	-0.002	0.030***	0.040***	0.011	0.118***	0.137***	0.141***	
Mf	0.018**	0.024***	0.009	0.001	0.022	0.033***	0.036*	0.114***	0.136***	0.138***	
Mt		-0.002	-0.003	-0.008	0.016***	0.019**	0.013	0.105***	0.133***	0.133***	
Pe	0.016		-0.003	0.001	0.022***	0.023***	0.027	0.108***	0.130***	0.135***	
Na	0.007	0.006		-0.009	0.013***	0.007	0.019	0.103***	0.126***	0.128***	
To	-0.045	-0.047	-0.053		-0.006	0.001	0.002	0.071***	0.094***	0.095***	
NB	0.011	-0.002	0.001	-0.059		0.012	0.001	0.070***	0.094***	0.098***	
Tu	0.002	-0.008	-0.001	-0.034	-0.001		0.025	0.105***	0.123***	0.126***	
SM	0.268***	0.117***	0.258***	0.167	0.217**	0.173***		0.093***	0.125***	0.131***	
Pa	0.522***	0.454***	0.501***	0.306***	0.453***	0.423***	0.302***		0.010	0.013	
PS	0.522***	0.446***	0.504***	0.318***	0.457***	0.431***	0.307***	0.009		0.006	
Ka	0.473***	0.392***	0.457***	0.254***	0.406***	0.379***	0.250***	-0.045	0.008		

Significance only accepted when 95% confidence interval > 0 (S2 Table). Site codes as in Table 1. Significance levels:

* p<0.0241;

** p<0.01;

*** p<0.001. Non-significant if nothing is indicated.

**Table 4:** ϕ_ct_- and F_ct_–values of hierarchical AMOVAs based on mtDNA (CR) and microsatellite data of *Amphiprion akallopisos* populations from the Indian Ocean across potential oceanographic barriers to dispersal.

Groupings	Barrier(s)	ϕ_ct_-value	SL	F_ct_-value	SL
(EIO) (WIO)	1	0.620	***	0.114	***
(EIO) (La, Wa) (Mo, Di, Ki, Mi, St, Dw, Ja, DS, Mf, Mt) (Pe, Na, To) (Tu, NB, SM)	1, 2, 3, 4	0.289	***	0.044	***
(La, Wa) (Mo, Di, Ki, Mi, St, Dw, Ja, DS, Mf, Mt) (Pe, Na, To) (Tu, NB, SM)	2, 3, 4	0.008	NS	0.007	**
(La, Wa) (Mo, Di, Ki, Mi, St, Dw, Ja, DS, Mf, Mt) (Pe, Na, To, Tu, NB, SM)	2, 3	0.006	NS	0.004	*
(La, Wa, Mo, Di, Ki, Mi, St, Dw, Ja, DS, Mf, Mt) (Pe, Na, To) (Tu, NB, SM)	2, 4	0.011	*	0.006	*
(La, Wa) (Mo, Di, Ki, Mi, St, Dw, Ja, DS, Mf, Mt, Pe, Na, To) (Tu, NB, SM)	3, 4	0.010	NS	0.013	***
(La, Wa, Mo, Di, Ki, Mi, St, Dw, Ja, DS, Mf, Mt) (Pe, Na, To, Tu, NB, SM)	2	0.009	NS	0.002	NS
(La, Wa) (Mo, Di, Ki, Mi, St, Dw, Ja, DS, Mf, Mt, Pe, Na, To, Tu, NB, SM)	3	-0.004	NS	0.006	NS
(La, Wa, Mo, Di, Ki, Mi, St, Dw, Ja, DS, Mf, Mt, Pe, Na, To) (Tu, NB, SM)	4	0.017	NS	0.014	**

Barrier(s): groups composed of sites on either side of one or more potential oceanographic barriers. 1: open ocean between EIO and WIO; 2: split of the SEC at approximately 10° S; 3: Convergence of EACC and SC; 4: Mozambique Channel Eddies (see also Fig 1). Site codes as in Table 2. SL: Significance levels:

*: p < 0.05;

**: p < 0.01;

***: p < 0.001; NS: non-significant.

The results from the Migrate analysis confirmed the very limited gene flow between the WIO and the EIO. The 95% posterior distribution interval for migration in both directions between WIO and EIO overlapped with 0. The distribution interval gives the probability that the actual value of the assessed parameter falls within the interval. Within the WIO, 95% posterior distribution also overlapped with 0, except for estimates of migration from West-Madagascar to East-Madagascar. In the EIO, gene flow was equal and relatively high in both directions (Table 5).

**Table 5:** Bayesian estimates of migration rates among populations of *Amphiprion akallopisos* using the software Migrate.

Control region	Mean	PD (2.5%-97.5%)	Microsatellites	Mean	PD (2.5%-97.5%)
Direction			Direction		
WIO → EIO	70.6	0.0–152.7	WIO → EIO	125.6	97.6–160.0
EIO → WIO	5.2	0.0–20.7	EIO → WIO	37.1	15.5–51.2
Eafr → Mad	211.7	0.0–399.3	Eafr → Mad	159.0	137.2–173.0
Mad → Eafr	574.2	0.0–869.0	Mad → Eafr	777.8	763.0–880.0
StM → WMad	135.3	0.0–370.0	StM → WMad	726.0	711.0–743.0
WMad → StM	660.3	278.0–1000.0	WMad → StM	277.0	257.0–297.0
Pad → JavSea	224.2	0.0–542.0	Pad → JavSea	175.7	144.0–207.5
JavSea → Pad	656.3	317.3–992.0	JavSea → Pad	174.1	142.4–202.7

Site codes: WIO: Western Indian Ocean; EIO: Eastern Indian Ocean; Eafr: East African coast; Mad: Madagascar; StM: Sainte Marie; WMad: Nosy Bé and Tuléar; Pad: Padang; JavSea: Karimun Java and Pulau Seribu. Mean: mean estimated number of migrants per generation; PD: 2.5%-97.5% posterior distribution of estimated migration rates.

#### Microsatellites

The same False Discovery Rate threshold (p<0.0241) as for mtDNA analysis was applied, and F_st_—values significantly different from 0 were equally checked for 95% confidence interval overlap with 0 (S1 Table). Differentiation between WIO and EIO populations was also detected with microsatellite markers. Hierarchical AMOVA grouping WIO against EIO yielded a significant F_ct_-value (F_ct_ = 0.11; p < 0.001; Table 4) and all pairwise F_st_-values between WIO and EIO populations were also significant, ranging from 0.070 to 0.151 (Table 3). This was confirmed by a Principal Component Analysis (PCA), where the first axis clearly distinguished the EIO populations from the WIO populations (Fig 1d). Low levels of gene flow between both regions were also estimated with the software Migrate, although the 95% posterior distribution was higher than 0, with a higher migration from the WIO towards the EIO (Table 5).

The highest ΔK in Evanno’s test was reached for K = 2 (ΔK = 1312.862). In a STRUCTURE analysis conducted under this scenario the WIO populations were almost exclusively assigned to cluster one and EIO populations almost exclusively assigned to cluster two (Fig 1e). When a DAPC was conducted without prior definition of clusters, but forcing the dataset into 2 clusters, the EIO individuals were also clearly separated from the WIO individuals on the first axis (Fig 1d). These results form a strong indication of the pronounced genetic differentiation between the EIO and the WIO populations.

Among WIO populations, a shallow but significant population structure was detected (overall F_st_ = 0.012; p < 0.001). Three distinct groups could be derived from the pairwise F_st_-values. One population from the North of Kenya, Watamu, had a high number of significant pairwise F_st_-values (12/17 pairwise comparisons) with the other populations of the WIO, as well as the three populations from Madagascar: Nosy Bé, Tuléar and Sainte Marie (9/17, 7/17 and 2/17, respectively; Table 3). This suggests the existence of three geographically distant, differentiated groups which are separated by oceanographic barriers 3 and 4. Hierarchical AMOVA confirmed differentiation between populations on either side of barrier 4, between Madagascar and East-Africa, but not of barrier 3, between northern Kenya and populations further South (Table 4).

Within both the WIO and EIO regions, relatively high values of migration were measured in all directions with Migrate (Table 5). Within the WIO, estimated levels of migration were threefold higher from East to West than in the other direction (Table 5).

Under Evanno’s test, K = 2 had the highest ΔK (542.896) followed by K = 3 (89.823). When a STRUCTURE analysis was conducted with both scenarios, however, clustering of individuals did not correspond to two or three geographically distinct areas, but rather failed to assign individuals to either of the two or three clusters (S1 Fig).

In PCA analysis, individuals belonging to the Northern Kenyan populations (Watamu and Lamu), Madagascar, and the rest of the WIO populations, were not differentiated (S2 Fig). However, a DAPC, designed to optimise differentiation among individuals, did distinguish between Malagasi and East African populations when two clusters were set out on a single axis with Malagasi individuals mostly clustering within the same group. Such a distinction was not detected between the population of Northern Kenya (Watamu and Lamu) and the rest of the WIO populations (S3 Fig).

### Assessment of limited sample size bias

In the two datasets with reduced sample size we composed and tested, the number of pairwise ϕ_st_-values that were significantly different from 0 dropped from 81 to 56 (69%) and 55 (68%) in Control Region analysis (S3A Table). The number of pairwise F_st_-values in microsatellite analysis that was significantly different from 0 dropped from 108 to 76 (70%) and 64 (59%) in the reduced datasets (S3B Table). Furthermore, ϕ/F_st_-values were on average lower when the reduced datasets were used, and the difference between reduced dataset values and complete dataset values was not significantly different from 0 (S3A and S3B Table). Based on the Control Region sequences, the population structure derived from AMOVA was similar in the reduced datasets, with lower ϕ_ct_-values (S3D Table). In the microsatellite analysis, F_ct_-values were also lower in the reduced datasets, and the differentiation between the population from Watamu and the rest of the WIO was no longer supported, with 95% Confidence Intervals overlapping 0 and non-significant F_ct_-values. Genetic differentiation between the EIO and WIO, and between Malagasy and East-African populations, was still detected (S3E Table).

Genetic diversity was not higher in the populations of which we analysed only small sample sizes than in the populations in which more samples were analysed (Table 1). We therefore find no indication that the populations in our dataset for which a relatively small (less than 20) number of samples were analysed contain an excess number of rare alleles that could distort our assessment by artificially inflating differentiation among populations. Rather than increasing the risk of false positive results, a reduced sample size in our dataset seems to cause a decrease in differentiation among populations. The most plausible explanation for this is that decreasing the sample size simply decreases genetic diversity.

## Discussion

### EIO-WIO genetic break confirmed

Genetic differentiation between the WIO and EIO populations of the skunk clownfish, already demonstrated with mtDNA [9], was confirmed with microsatellite markers. These findings are congruent with earlier studies on algae, fishes, and invertebrates that indicate limited gene flow between the EIO and the WIO [7, 8, 10]. Both the F_st_-values derived from AMOVA and the pairwise F_st_-values from the microsatellite analysis were two to four times lower than the same values derived from mtDNA analysis. This is not surprising and can be attributed to two separate phenomena. First, because mtDNA is maternally inherited, effective population size is smaller, which results in higher genetic drift and faster differentiation among populations [26]. Second, within population diversity is relatively high when measured with microsatellite markers compared to mtDNA and this deflates F_st_-values, without therefore affecting their significance [57].

Nuclear and mitochondrial markers were also congruent in detecting higher genetic diversity in the EIO compared to the WIO. Higher genetic diversity in the EIO in combination with the fact that EIO and WIO individuals do not form separate clades in our haplotype network supports the hypothesis that the EIO and WIO populations are composed of descendants from a pan-Indian Ocean population that originated in the EIO [9]. The haplotype network forms multiple, reticulate, links between the WIO and EIO groups that somewhat contradict the overall results of high differentiation between both regions. We think, however, that these reticulate links are merely artefacts caused by the high genetic diversity in the EIO population. Within the latter, the haplotype network contains a high number of mutational steps between haplotypes. It is possible that some of these mutational steps are actually intermediate haplotypes we have not been able to sample or which became extinct. Only additional sampling in the EIO could solve this issue. Another possibility for the rather unusual haplotype network could be that a former population in the central Indian Ocean became extinct and intermediate haplotypes between the WIO and EIO haplotypes got lost.

### Population structure in the WIO

#### East African mainland versus Madagascar

There was a sign of genetic differentiation between Malagasy populations on one side and African mainland populations on the other side. These results are congruent with those of two other studies in the WIO that also detected genetic differentiation between populations on both sides of the Mozambique Channel in a soldierfish [17] and a mangrove crab [18], and a third study where genetic differentiation between populations on the Mascarene Plateau and eastern Madagascar on one hand and populations within the Mozambique Channel and in East Africa on the other hand was discovered in a grouper [58]. They do not concur with studies on the blue starfish [10], the Dory snapper [19], the Kashmir snapper [22], and the crown-of-thorns starfish [21], where no genetic differentiation between populations on both sides of the Mozambique Channel was detected. It is, however, difficult to compare the results of these studies, because samples were collected at different sample sites and often only partly cover the Mozambique Channel. Furthermore, not all of these studies use identical genetic markers. In the future, a more integrated approach among research groups, using identical sampling sites and genetic markers across species when possible, would greatly improve our insight in marine connectivity in the WIO.

The Mozambique Channel sea surface currents are characterised by a series of anticyclonic mesoscale eddies interspersed by smaller cyclonic eddies that move water slowly southward through the channel and are capable of trapping particles for several months [59], creating an effective barrier to gene flow between Madagascar and the African mainland. However, frontal zone transport also seems to exist between the edges of these eddies, occasionally creating connectivity between Madagascar and the African mainland. Experimental drifters have been recorded to cross the Mozambique Channel using these frontal zones in as little as 15 days [59], which corresponds exactly to the estimated PLD of *A*. *akallopisos* larvae [60]. Such a combination of overall larval retention mediated by mesoscale eddies preventing continuous gene flow and occasional connectivity through frontal zone transport offsetting complete differentiation may perfectly explain the pattern of moderate genetic differentiation we encountered. In such a scenario, gene flow from Madagascar towards the East African coast would be more frequent than in the other direction, which corresponds to our findings. Geographically intermediate populations of skunk clownfish occur on several islands and atolls in the Mozambique Channel. Recently, significant population structure was detected among populations of the skunk clownfish on four of these islands, indicating two barriers to gene flow for this species within the Mozambique Channel, separating northern, central, and southern populations. The complex oceanography and more specifically eddies in the Mozambique Channel were suggested as causes for these barriers to gene flow [61]. The genetic relatedness of these island populations with the populations studied here, however, is not known. However, populations of several other reef fishes did not show significant genetic structure across [17, 22, 58] or within [61] the Mozambique Channel.

Both marker types detected genetic differentiation across the Mozambique Channel, but mtDNA only identified the Sainte Marie population in East Madagascar as differentiated, whereas microsatellites indicated genetic differences among Madagascar and the African mainland. Furthermore, with both marker types there was an indication of stronger gene flow from Madagascar to the East African coast than in the other direction, but in mtDNA the signal was restricted to reduced gene flow from eastern Madagascar to the West. One possible explanation for the discordance in this context is the higher resolving power of several microsatellite markers combined over mtDNA as a single locus [29]. The Sainte Marie population is separated from the East African mainland by a larger geographic distance than the other Malagasy populations included in this study. In addition to this, larval dispersal from Sainte Marie to the other reefs may not only be hampered by the sea surface current configuration in the Mozambique Channel as described earlier, but further restricted by the SEC that splits into the NEMC and the SEMC at approximately the location of Sainte Marie, i.e. 17° South [13]. Therefore, Sainte Marie might be isolated more strongly from the East African coast than the populations from West Madagascar and mtDNA markers might be able to detect this signal of genetic differentiation along with microsatellites, but not the weaker signal between western Madagascar and East Africa.

However, after corrections for false discovery and confidence interval uncertainty, Sainte Marie was only significantly differentiated from two African mainland populations (Stone Town and Mafia) based on microsatellites. This could be a consequence of the low sample size at this site. As illustrated with tests using a reduced number of samples for all populations (S3A and S3B Table), a lower sample size seems to decrease statistical power in our dataset. With microsatellite markers, where intra-population diversity can be high when many alleles exist per locus, the loss of statistical power can be more explicit than with less variable markers like mtDNA [53].

Another possible explanation can be derived from the fact that mtDNA is maternally inherited. The skunk clown fish, like all anemone fishes, lives in size-based hierarchical groups with only one female per group of four to eight individuals [62, 63]. Effective population size is therefore strongly reduced for mtDNA, which can cause founder effects and promotes genetic drift in an isolated population like Sainte Marie. Yet another explanation could be that repetitive microsatellite sequences evolve more rapidly than mtDNA and therefore the latter provides a more historical picture of gene flow. Estimates of migration rates suggest lower levels of migration based on mtDNA than on microsatellites, both between East Africa and Madagascar and among Malagasy populations. This could indicate that connectivity in the WIO has changed over time and both marker types reflect gene flow of a different time frame. In any case, this discordance between mtDNA and microsatellites further highlights the need to combine both marker types to obtain an optimal estimate of gene flow among populations of marine organisms [32].

#### Northern Kenya versus southern regions

Contrary to the mtDNA analysis, pairwise F_st_-values based on microsatellite markers suggested a moderate genetic break between the North Kenyan population of Watamu and other populations in the WIO. Geographically, genetic differentiation between the population at Watamu and populations further South corresponds to a suggested barrier to larval dispersal mediated by the confluence of the Somali Current and the EACC (barrier 3), which can prevent larval dispersal across this zone [13, 16, 17]. However, only the population of Watamu was differentiated from the rest, and not the population of Lamu, located further to the North of Watamu. If sea surface currents form a barrier to dispersal in South to North direction along the Kenyan coast, then we would expect to see differentiation between Lamu and more southern populations as well, but hierarchical AMOVA did not support this hypothesis. The genetic differentiation between Watamu and other populations could also be explained by the stochastic nature of larval dispersal, which could have created a pattern of chaotic genetic patchiness that can be responsible for the emergence of genetic differentiation among populations [64]. Genetic differentiation between Watamu and other populations, however, was not inferred by the analyses based on confidence intervals and clustering methods. When sample sizes were reduced in all populations, genetic differentiation was also no longer detected between Watamu and the other populations. Overall, statistical power dropped to a level that no longer permitted the detection of population structure within the WIO with a sample size of only eight individuals per population. This could suggest that population structure might be present in the WIO, but could not be detected due to the limited number of analysed samples in some populations. Only the analysis of additional samples from these sites could provide an answer to this question.

## Conclusions

This study confirms earlier findings on a barrier to gene flow and larval dispersal separating the WIO and EIO populations of the skunk clownfish. It also identifies fine scale population structure within the WIO. Two of the three suggested current-mediated barriers to larval dispersal, the split of the SEC at approximately 10° S, and the convergence of the Somali Current with the EACC at approximately 3° S, were not found to form a barrier to gene flow in this species. The third, caused by the complex oceanographic nature of the Mozambique Channel, could promote genetic isolation among Malagasy and African mainland populations. The exact geographic location of this differentiation, however, shifted depending on the genetic markers used. Estimated gene flow within the WIO was found to be stronger from East to West than in the other direction, which corresponds to the direction of the dominant sea surface current, but was also different between marker types. The latter could be an indication of variability of gene flow over time. Furthermore, significant population structure has been detected among populations of the skunk clownfish within the Mozambique Channel, confirming the possible restricting influence of eddies on gene flow in the Mozambique Channel, at least in some reef fish species [61].

## Supporting information

S1 Table Overview of microsatellite markers tested for *A*. *akallopisos*. (DOCX)

S2 Table 95% Confidence Intervals ϕ_st_ and F_st_-values. (DOCX)

S3 Table (A) Pairwise ϕst-values among populations using reduced datasets. (B) Pairwise Fst-values among populations using reduced datasets. (C) Composition of reduced populations with eight randomly chosen individuals. (D) ϕct–values of hierarchical AMOVAs. (E) Fct–values of hierarchical AMOVAs.
(DOCX)

S1 Fig Structure k = 3 for WIO. (DOCX)

S2 Fig PCA of the WIO using 3 predefined groups. (DOCX)

S3 Fig DAPC of WIO individuals defining 2 clusters. (DOCX)
