# Supplementary material for: Sea surface currents and geographic isolation shape the genetic population structure of a coral reef fish in the Indian Ocean
Source: PLoS One. 2018 Mar 9;13(3):e0193825. doi: 10.1371/journal.pone.0193825 (PMC5844546; doi:10.1371/journal.pone.0193825)
Supplement: S1 Table — (DOCX) [file pone.0193825.s001.docx]

**S1 Table. Overview of microsatellite loci tested in *Amphiprion akallopisos****.* Name of the locus, forward and repeat primer sequences, the repeat motive, number of alleles in the test-population, maximum and minimum length of the repeat, or average length when only this information is available, the species in which they were tested, and the publication in which the information appeared. Full publication references under the table. Markers retained and used in final analysis are indicated in grey.

| **Locus** | **Primer sequences** | **Repeat motive** | **alleles** | **Repeat length** | **Species** | **Publication** |
| --- | --- | --- | --- | --- | --- | --- |
| Cf4 | F-CAGAGTGTGCAGCGAGTAG  R-CGTCCATAGAAGGTAAGGAG | ACAG | 2 | 187-196 | A. percula | Buston et al. 2007 |
| Cf8 | F-CTGTTAGTATTCATCCATTTTCTTTG  R-TTTTTACTCTGTCTTTGTGGGTTTAT | ATAG | 2 | 296-300 | A. percula | Buston et al. 2007 |
| Cf12 | F-CATGGGAGCCAAATGTAAGAAT  R-TGTCACTTTATCCTGCAACCCAGAT | AC | 8 | 221-246 | A. percula | Buston et al. 2007 |
| Cf21 | F-CACAGCAGAGTTTTGGATGG  R-AAAAGACGAAGGGAGTAAGTG | AAAG | 14 | 148-192 | A. percula | Buston et al. 2007 |
| Cf29 | F-TTCTTTATCCCCTTGTTTATTTCTAA  R-AAGCCTCCTTTCCAAAACCACTCA | AC | 24 | 244-302 | A. percula | Buston et al. 2007 |
| Cf39 | F-CCGGACAGCCAGAGCAAAGA  R-CCTAATCGATCGGTGGTGACAT | AGAC | 17 | 317-387 | A. percula | Buston et al. 2007 |
| Cf42 | F-AAGCTCCGGTAACTCAAAACTAAT  R-GTCATCTGATCCATGTTGATGTG | AGAC | 9 | 305-346 | A. percula | Buston et al. 2007 |
| 3GATA | F-TCAGGCTGTCCTGCAGACTCAGG  R-ATGTTGAAATACAGCAACCTACA | GATA | 17 | 386 | A. polymnus | Quenouille et al. 2004 |
| 10TCTA | F-GGGACGTATCTGTTGGAAATGAT  R-TTAAGGTACTGTGAGATGAGACT | TCTA | 19 | 544 | A. polymnus | Quenouille et al. 2004 |
| 61 | F-TGAACACATAAACGCTCACTCAC  R-AAGACAATGCCTCCACATATCTA | GT | 20 | 345 | A. polymnus | Quenouille et al. 2004 |
| 79 | F-GCATGGATGGTCACAGAGGAGCT  R-GGAGCATTCGCCACTCTTCATAT | GT | 11 | 191 | A. polymnus | Quenouille et al. 2004 |
| 45 | F-TCAACTGAATGGAGTCCATCTGG  R-CCGCCGCTAGCCGTGACATGCAA | GT | 10 | 264 | A. polymnus | Quenouille et al. 2004 |
| 120 | F-TCGATGACATAACACGACGCAGT  R-GACGGCCTCGATCTGCAAGCTGA | GT | 8 | 489 | A. polymnus | Quenouille et al. 2004 |
| 2 | F-TTGCATTACATTAATCAGTGCT  R-CTTGATGACTTGTTCTGGCT | GT | 6 | 219 | A. polymnus | Quenouille et al. 2004 |
| 44 | F-TTGGAGCAGCGTACTTAGCT  R-AGATGTGTTTACGCACGCTT | GT | 11 | 295 | A. polymnus | Quenouille et al. 2004 |
| 55 | F-TTAACTTCCACACCCAGTCT  R-ACGCTGTGAGAGTCCATTAT | GT | 16 | 438 | A. polymnus | Quenouille et al. 2004 |
| 22 | F-TCTGTTCACTTTATCCAACTTGT  R-GGAGACACCAGGCCAATAGA | GT | 30 | 376 | A. polymnus | Quenouille et al. 2004 |
| 65 | F-AGGCCGTGAGTAACTACATTGTT  R-GGAGGTGTGGCTGCCATGCCTGT | GT | 9 | 258 | A. polymnus | Quenouille et al. 2004 |
| Cf9 | F- CTCTATGAAGATTTTT  R- GTACATGTGTTTCCTC | TCAA | 18 | 229-301 | A. ocellaris | Timm et al. 2012 |
| Cf27 | F- AAGCTCCGGTAACTCAAAACTAAT  R- GTCATCTGATCCATGTTGATGTG | TCTA | 34 | 171-301 | A. ocellaris | Timm et al. 2012 |
| AC1359 | F- TGAAATACGATGCGGAAACA  R-GAAGGCGAATACGTGGGATA | AC | 6 | 242 | A. clarkii | Liu et al. 2007 |
| AC1578 | F- CAGCTCTGTGTGTGTTTAATGC  R- CACCCAGCCACCATATTAAC | AC | 5 | 251 | A. clarkii | Liu et al. 2007 |
| AC915 | F- TTGCTTTGGTGGAACATTTGC  R- TCTGCCATTTCCTTTGTTC | AC | 7 | 227 | A. clarkii | Liu et al. 2007 |
| AC137 | F- GGTTGTTTAGGCCATGTGGT  R- TTGAGACACACTGGCTCCT | AC | 29 | 273 | A. clarkii | Liu et al. 2007 |
| AC277 | F- TTTGCTCTTGTGGGGAGTTC  R- TGTTCTCTAGTGGGGGTGGA | AC | 24 | 226 | A. clarkii | Liu et al. 2007 |
| AC522 | F- GCTTTCAATGGACCTGTGCT  R- TGGCATAACTTCAGTTTGTTGC | AC | 29 | 203 | A. clarkii | Liu et al. 2007 |
| AC626 | F- CACACATGCACACACCTTGA  R- TAATTGAGGCAGGTGGCTTC | AC | 21 | 230 | A. clarkii | Liu et al. 2007 |
| AC1240 | F- TAACACAGCCCTGCACATTC  R- ATTGGTGCGAGGACAACTCT | AC | 30 | 282 | A. clarkii | Liu et al. 2007 |
| B6 | F-TGTCTTCTCCCCAAGTCAG  R-ACGAGGCTCAACATACCTG | CATC | 14 | 141-159 | A. clarkii | Pinsky et al. 2010 |
| C1 | F-GCGACCTTGTTATCACTGTC  R-TTGGTTGGACTTTCTTTGTC | CATC | 8 | - | A. clarkii | Pinsky et al. 2010 |
| LIST12_004 | F-CAGTATTAGGCAGGAGGTGG  R-CTGACACACAGAGAGACAGC | TG | 3 | 255-307 | A. clarkii | Watts et al. 2004; Pinsky et al. 2010 |
| LIST12_012 | F-GATTGATACATACAGCACCGC  R-AAGTCTGACACTACCGCAGG | TG | 4 | 119-191 | A. clarkii | Watts et al. 2004; Pinsky et al. 2010 |
| LIST12_028 | F-GGGCTGTTTATGTCAGTTTAGC  R-CTAATGCGGTGTTTCCATCCC | AC | 2 | 283-285 | A. clarkii | Watts et al. 2004; Pinsky et al. 2010 |
| D1 | F-CCAAAAGTTTAGGAAGCTACC  R-AACCAGACTGCCCTGATAC | GATA | 13 | 273-341 | A. chrysopterus | Beldade et al. 2009 |
| A115 | F-GACTCGTGTTCGGAGGAC  R-CGGGATAATAACGGAGAGC | CA | 12 | 212-242 | A. chrysopterus | Beldade et al. 2009 |
| A130 | F-GCACTCAACACAAAGACCTTA  R-ACCCAAACAACATCCAGTC | CA | 12 | 160-194 | A. chrysopterus | Beldade et al. 2009 |
| A131 | F-CCTCAGCAGTGTGAAATGA  R-CTCCACCTCTCTCTTCTTGAC | CA | 13 | 200-250 | A. chrysopterus | Beldade et al. 2009 |
| CF11 | F-CTCCACCTCTCTCTTCTTGAC  R-GTAATTGCTGCAAGACAG | CA | 8 | 180-206 | A. chrysopterus | Beldade et al. 2009 |
| D103 | F-GTTGGCTAATGGTGCTGTG  R-GATTCTGTGGTGGCATCAG | GATA | 4 | 239-251 | A. chrysopterus | Beldade et al. 2009 |
| D108 | F-GAAGGATGTGCTTTGTTGTTC  R-GCTTTACGATTTTACAATGCAC | GATA | 14 | 286-338 | A. chrysopterus | Beldade et al. 2009 |
| D114 | F-TGTTCCAGCTCTGATATTTGAC  R-TTGGCAGTGTTTTATACCTGTC | GATA | 11 | 205-285 | A. chrysopterus | Beldade et al. 2009 |
| Am1 | F- ACAAAGCCTTCATGTGGGTC  R- CGCAAGTGTTGCCTCATAGA | TG | 2 | 108-112 | A. mccullochi | Van der Meer et al. 2012 |
| Am4 | F-AGCTGGTTGGGTGTTACCTG  R-ATGGCTTCAGTCTGCTGGTT | GT | 11 | 128-156 | A. mccullochi | Van der Meer et al. 2012 |
| Am5 | F-CCACTAGAGGCTCCCTGTTG  R-CGAGCATGAGCGAATGTATG | AC | 9 | 77-95 | A. mccullochi | Van der Meer et al. 2012 |
| Am6 | F-AGCAGAGAGGAAAGAAGGGC  R-CAAGTGCCTGGCAGAAGATT | TGT | 4 | 262-274 | A. mccullochi | Van der Meer et al. 2012 |
| Am7 | F-TGTCGCTACGACAGACTGCT  R-GCATGAGTGATTGGACCCTA | ATG | 10 | 86-128 | A. mccullochi | Van der Meer et al. 2012 |
| Am9 | F-TGCTGCACTCTGTCTATTTTGT  R-GTGACTGAAGGCAAGGCAAT | TTA | 13 | 151-202 | A. mccullochi | Van der Meer et al. 2012 |
| Am10 | F-GGAAGCAGCAATAAAGACGC  R-AGAGACGCCTGATGGTGAGT | ACAG | 5 | 286-302 | A. mccullochi | Van der Meer et al. 2012 |
| Am11 | F-ATTCCCCGACGGAGAGTAGT  R-TGTCGCTTTGTGACACCTTC | CTAT | 7 | 124-172 | A. mccullochi | Van der Meer et al. 2012 |
| Am12 | F-ATGAGCAGCTTTGACGGAAT  R-ACCTACATGGTTGGAGCCTG | TTCA | 8 | 182-210 | A. mccullochi | Van der Meer et al. 2012 |
| Am14 | F-CAGCAGCCTCAAGTGACTGT  R-GCAGCATTCTCACACACCAC | GATG | 8 | 170-198 | A. mccullochi | Van der Meer et al. 2012 |
| Am15 | F-ACTAGGCTCAGAGCAGGGTC  R-CAAGTCAATCAAAGCAGCCA | GTCT | 11 | 100-160 | A. mccullochi | Van der Meer et al. 2012 |
| Am17 | F-GGCTGTCTGGGATGAGATGT  R-TGTTCTGCAGATGGACTGTTTT | AATA | 9 | 105-138 | A. mccullochi | Van der Meer et al. 2012 |
| Am18 | F-TGGTCCTAGCAGCTGTCTGT  R-GGCTACATCTGCAACGACAA | TGAA | 8 | 87-119 | A. mccullochi | Van der Meer et al. 2012 |
| Am19 | F-CTGTAATGAATCCAAGGAGCTG  R-TGGATAATGAAGAAATGGATGG | TCCA | 13 | 102-146 | A. mccullochi | Van der Meer et al. 2012 |
| Am21 | F-TCTCGTCTGGTGTTGACTGC  R-CAATGGCTTTACTTTTCTCTGC | TTCTA | 9 | 95-140 | A. mccullochi | Van der Meer et al. 2012 |
| Am22 | F-GCCGAATATGCCGTACAACT  R-TATCTTCAGACCCACCTGGC | TGGGTC | 10 | 110-155 | A. mccullochi | Van der Meer et al. 2012 |
| Am24 | F-CTGCTGGATCAGGGTTAGGA  R-ACCATGCCCAGGTACTGTCT | TCAGGA | 4 | 141-165 | A. mccullochi | Van der Meer et al. 2012 |

**Beldade, R.**, Holbrook, S.J., Schmitt, R.J., Planes, S., Bernardi, G. (2009) Isolation and characterization of eight polymorphic microsatellite markers from the orange-fin anemonefish *Amphiprion chrysopterus*, *Conservation Genetics Resources*, 1, 333-335.

**Buston, P.M.**, Bogdanowicz, S.M., Wong, A., Harrison, R.G. (2007) Are clownfish groups composed of close relatives? An analysis of microsatellite DNA variation in *Amphiprion percula*, *Molecular Ecology*, 16, 3671-3678.

**Liu, S.V.**, Yu, H.T., Dai, C.F. (2007) Eight microsatellite loci in Clark’s anemonefish *Amphiprion clarkii*, *Molecular Ecology Notes*, 4, 1169-1171.

**Pinsky, M.L.**, Montes, H.R.Jr., Palumbi, S.R. (2010) Using isolation by distance and effective density to estimate dispersal distances in anemonefish, *Evolution*, 64, 2688-2700.

**Quenouille, B.**, Bouchenak-Khelladi, Y., Hervet, C., Planes, C. (2004) Eeleven microsatellite loci for the saddleback clownfish *Amphiprion polymnus*, *Molecular Ecology Notes*, 4, 291-293.

**Timm, J.,** Planes, S., Kochzius, M. (2012) High similarity of genetic population structure in the False Clown Anemonefish (*Amphiprion ocellaris*) found in microsatellite and mitochondrial control region analysis, *Conservation Genetics*, 13, 693-706.

**Van der Meer, M.H.**, Gardner, M.G., Hobbs, J-P.A., Jones, G.P., van Herwerden, L. (2012) Identification of seventeen microsatellite markers for conservation genetic studies of the endemic anemonefish, *Amphiprion mccullochi*, *Conservation Genetics Resources*, 4, 247-250.

**Watts, P.C.**, Veltsos, P., Soffa, B.J., Gill, A.B., Kemp, S.J. (2004) Polymorphic microsatellite loci in the black-and-gold chromis, *Neoglyphidodon nigroris* (Teleostei: Pomacentridae), *Molecular Ecology Notes*, 4, 93-95.
