# Supplementary material for: Sea surface currents and geographic isolation shape the genetic population structure of a coral reef fish in the Indian Ocean
Source: PLoS One. 2018 Mar 9;13(3):e0193825. doi: 10.1371/journal.pone.0193825 (PMC5844546; doi:10.1371/journal.pone.0193825)
Supplement: S2 Table — (DOCX) [file pone.0193825.s002.docx]

**S2 Table. 2.5% - 97.5% Confidence Intervals** for φ_st_-values (mtDNA) and F_st_-values (Microsatellites) of pairwise comparisons of populations of *Amphiprion akallopisos* that are significantly different from 0 in Arlequin. Significance: *: p<0.05; **: p<0.01; ***: p<0.01. Pop comparison: populations compared. Site letter codes as in Table 1.

| **Pop comparison** | **Φ_ST_-value** | **2.5-97.5%-Confidence Interval** |
| --- | --- | --- |
| Ki-Mo | 0.096* | 0.002 – 0.125 |
| Ki-Di | 0.079* | -0.011 – 0.104 |
| Ki-Dw | 0.087*** | 0.001 – 0.121 |
| Ki-Ds | 0.124*** | 0.008 – 0.142 |
| Ki-Mt | 0.064** | -0.019 – 0.134 |
| Ki-Na | 0.060** | -0.026 – 0.124 |
| Ki-Nb | 0.072*** | 0.000 – 0.121 |
| Ki-Tu | 0.056* | -0.004 – 0.101 |
| Ds-La | 0.152* | -0.023 – 0.447 |
| Ds-Wa | 0.017* | 0.003 – 0.046 |
| Pe-Mt | 0.016* | -0.013 – 0.022 |
| Sm-La | 0.164* | -0.027 – 0.216 |
| Sm-Wa | 0.191*** | 0.004 – 0.264 |
| Sm-Mo | 0.271*** | 0.030 – 0.312 |
| Sm-Di | 0.235*** | 0.009 – 0.274 |
| Sm-Ki | 0.188*** | 0.013 – 0.247 |
| Sm-Mi | 0.182*** | -0.003 – 0.257 |
| Sm-St | 0.262*** | 0.025 – 0.319 |
| Sm-Dw | 0.258*** | 0.026 – 0.307 |
| Sm-Ja | 0.196* | -0.001 – 0.247 |
| Sm-Ds | 0.310** | 0.044 – 0.336 |
| Sm-Mf | 0.234** | 0.013 – 0.282 |
| Sm-Mt | 0.268*** | 0.030 – 0.364 |
| Sm-Pe | 0.117*** | 0.002 – 0.178 |
| Sm-Na | 0.258*** | 0.023 – 0.325 |
| Sm-Nb | 0.217** | 0.031 – 0.260 |
| Sm-Tu | 0.173*** | 0.012 – 0.215 |
| Pa-La | 0.321*** | 0.109 – 0.416 |
| Pa-Wa | 0.391*** | 0.151 – 0.467 |
| Pa-Mo | 0.425*** | 0.176 – 0.469 |
| Pa-Di | 0.394*** | 0.149 – 0.466 |
| Pa-Ki | 0.355*** | 0.126 – 0.447 |
| Pa-Mi | 0.389*** | 0.149 – 0.471 |
| Pa-St | 0.455*** | 0.184 – 0.518 |
| Pa-Dw | 0.429*** | 0.165 – 0.497 |
| Pa-Ja | 0.355*** | 0.119 – 0.447 |
| Pa-Ds | 0.461*** | 0.189 – 0.514 |
| Pa-Mf | 0.405*** | 0.150 – 0.479 |
| Pa-Mt | 0.522*** | 0.240 – 0.562 |
| Pa-Pe | 0.454*** | 0.207 – 0.519 |
| Pa-Na | 0.501*** | 0.221 – 0.548 |
| Pa-To | 0.306*** | 0.102 – 0.384 |
| Pa-Nb | 0.453*** | 0.201 – 0.497 |
| Pa-Tu | 0.423*** | 0.167 – 0.491 |
| Pa-Sm | 0.302*** | 0.119 – 0.410 |
| Ps-La | 0.336*** | 0.095 – 0.457 |
| Ps-Wa | 0.393*** | 0.138 – 0.495 |
| **Pop comparison** | **Φ_ST_-value** | **2.5-97.5%-Confidence Interval** |
| Ps-Mo | 0.435*** | 0.158 – 0.499 |
| Ps-Di | 0.408*** | 0.132 – 0.507 |
| Ps-Ki | 0.366*** | 0.108 – 0.482 |
| Ps-Mi | 0.397*** | 0.129 – 0.504 |
| Ps-St | 0.468*** | 0.170 – 0.551 |
| Ps-Dw | 0.435*** | 0.158 – 0.521 |
| Ps-Ja | 0.369*** | 0.101 – 0.481 |
| Ps-Ds | 0.475*** | 0.172 – 0.550 |
| Ps-Mf | 0.421*** | 0.139 – 0.513 |
| Ps-Mt | 0.522*** | 0.219 – 0.586 |
| Ps-Pe | 0.446*** | 0.181 – 0.538 |
| Ps-Na | 0.504*** | 0.209 – 0.579 |
| Ps-To | 0.318*** | 0.089 – 0.416 |
| Ps-Nb | 0.457*** | 0.176 – 0.521 |
| Ps-Tu | 0.431*** | 0.150 – 0.524 |
| Ps-Sm | 0.307*** | 0.105 – 0.442 |
| Ka-La | 0.276*** | 0.070 – 0.394 |
| Ka-Wa | 0.342*** | 0.115 – 0.453 |
| Ka-Mo | 0.384*** | 0.146 – 0.441 |
| Ka-Di | 0.355*** | 0.110 – 0.454 |
| Ka-Ki | 0.313*** | 0.090 – 0.431 |
| Ka-Mi | 0.342*** | 0.107 – 0.453 |
| Ka-St | 0.420*** | 0.151 – 0.509 |
| Ka-Dw | 0.391*** | 0.136 – 0.487 |
| Ka-Ja | 0.318*** | 0.086 – 0.432 |
| Ka-Ds | 0.428*** | 0.157 – 0.505 |
| Ka-Mf | 0.369*** | 0.118 – 0.467 |
| Ka-Mt | 0.473*** | 0.203 – 0.542 |
| Ka-Pe | 0.392*** | 0.156 – 0.488 |
| Ka-Na | 0.457*** | 0.186 – 0.530 |
| Ka-To | 0.254*** | 0.064 – 0.347 |
| Ka-Nb | 0.406*** | 0.160 – 0.470 |
| Ka-Tu | 0.379*** | 0.132 – 0.475 |
| Ka-Sm | 0.250*** | 0.076 – 0.385 |
|  |  |  |
| **Pop comparison** | **F_ST_-value** | **2.5-97.5%-Confidence Interval** |
| La-Mf | 0.026* | -0.015 – 0.037 |
| La-Pe | 0.021* | -0.015 – 0.033 |
| La-Tu | 0.024* | -0.004 – 0.036 |
| La-Pa | 0.077*** | 0.060 – 0.126 |
| La-Ps | 0.101*** | 0.068 – 0.162 |
| La-Ka | 0.112*** | 0.071 – 0.154 |
| Wa-Mo | 0.031* | -0.002 – 0.045 |
| Wa-Di | 0.038*** | 0.022 – 0.076 |
| Wa-Ki | 0.049*** | 0.023 – 0.078 |
| Wa-St | 0.029** | 0.026 – 0.061 |
| Wa-Dw | 0.031*** | 0.026 – 0.063 |
| Wa-Ja | 0.040*** | 0.027 – 0.066 |
| Wa-Ds | 0.044*** | 0.021 – 0.086 |
| Wa-Mf | 0.059*** | 0.037 – 0.092 |
| **Pop comparison** | **F_ST_-value** | **2.5-97.5%-Confidence Interval** |
| Wa-Mt | 0.022** | 0.002 – 0.041 |
| Wa-Pe | 0.026*** | 0.002 – 0.047 |
| Wa-Na | 0.031*** | 0.021 – 0.059 |
| Wa-Nb | 0.037*** | 0.029 – 0.071 |
| Wa-Tu | 0.049*** | 0.035 – 0.087 |
| Wa-Pa | 0.080*** | 0.071 – 0.122 |
| Wa-Ps | 0.108*** | 0.084 – 0.165 |
| Wa-Ka | 0.113*** | 0.079 – 0.161 |
| Mo-Nb | 0.020* | 0.004 – 0.042 |
| Mo-Tu | 0.026*** | -0.001 – 0.038 |
| Mo-Pa | 0.098*** | 0.064 – 0.139 |
| Mo-Ps | 0.118*** | 0.074 – 0.173 |
| Mo-Ka | 0.126*** | 0.070 – 0.168 |
| Di-Mi | 0.019* | -0.013 – 0.070 |
| Di-Nb | 0.031*** | -0.000 – 0.054 |
| Di-Tu | 0.032*** | -0.006 – 0.057 |
| Di-Sm | 0.031* | -0.001 – 0.051 |
| Di-Pa | 0.098*** | 0.062 – 0.125 |
| Di-Ps | 0.124*** | 0.065 – 0.171 |
| Di-Ka | 0.124*** | 0.058 – 0.154 |
| Ki-Ds | 0.021* | -0.000 – 0.029 |
| Ki-Pe | 0.011* | -0.001 – 0.016 |
| Ki-Nb | 0.031*** | 0.004 – 0.065 |
| Ki-Tu | 0.041*** | 0.000 – 0.063 |
| Ki-Sm | 0.028* | -0.000 – 0.046 |
| Ki-Pa | 0.122*** | 0.081 – 0.158 |
| Ki-Ps | 0.143*** | 0.085 – 0.198 |
| Ki-Ka | 0.151*** | 0.082 – 0.180 |
| Mi-Ja | 0.018* | -0.012 – 0.027 |
| Mi-Mf | 0.033*** | -0.005 – 0.040 |
| Mi-Nb | 0.018* | 0.004 – 0.059 |
| Mi-Tu | 0.021* | -0.001 – 0.053 |
| Mi-Pa | 0.116*** | 0.073 – 0.156 |
| Mi-Ps | 0.141*** | 0.089 – 0.185 |
| Mi-Ka | 0.147*** | 0.079 – 0.184 |
| St-Mf | 0.015* | 0.002 – 0.038 |
| St-Nb | 0.019* | 0.001 – 0.036 |
| St-Tu | 0.014* | -0.003 – 0.032 |
| St-Sm | 0.045* | 0.011 – 0.078 |
| St-Pa | 0.097*** | 0.074 – 0.127 |
| St-Ps | 0.122*** | 0.086 – 0.164 |
| St-Ka | 0.122*** | 0.074 – 0.151 |
| Dw-Tu | 0.018* | 0.006 – 0.026 |
| Dw-Pa | 0.096*** | 0.064 – 0.126 |
| Dw-Ps | 0.115*** | 0.065 – 0.162 |
| Dw-Ka | 0.117*** | 0.063 – 0.151 |
| Ja-Mt | 0.016* | -0.007 – 0.018 |
| Ja-Pe | 0.018* | -0.004 – 0.022 |
| Ja-Pa | 0.101*** | 0.075 – 0.127 |
| Ja-Ps | 0.120*** | 0.078 – 0.164 |
| Ja-Ka | 0.118*** | 0.068 – 0.144 |
| **Pop comparison** | **F_ST_-value** | **2.5-97.5%-Confidence Interval** |
| Ds-Mf | 0.015* | -0.003 – 0.043 |
| Ds-Mt | 0.008* | -0.003 – 0.024 |
| Ds-Nb | 0.030*** | 0.007 – 0.046 |
| Ds-Tu | 0.040*** | 0.029 – 0.058 |
| Ds-Sm | 0.011* | -0.001 – 0.070 |
| Ds-Pa | 0.118*** | 0.085 – 0.148 |
| Ds-Ps | 0.137*** | 0.088 – 0.177 |
| Ds-Ka | 0.141*** | 0.083 – 0.173 |
| Mf-Mt | 0.018** | 0.001 – 0.023 |
| Mf-Pe | 0.024*** | 0.005 – 0.034 |
| Mf-Nb | 0.022*** | -0.001 – 0.054 |
| Mf-Tu | 0.033*** | 0.002 – 0.061 |
| Mf-Sm | 0.036* | 0.014 – 0.066 |
| Mf-Pa | 0.114*** | 0.079 – 0.153 |
| Mf-Ps | 0.136*** | 0.085 – 0.192 |
| Mf-Ka | 0.138*** | 0.079 – 0.178 |
| Mt-Nb | 0.016*** | 0.005 – 0.025 |
| Mt-Tu | 0.019** | 0.004 – 0.024 |
| Mt-Pa | 0.105*** | 0.071 – 0.133 |
| Mt-Ps | 0.133*** | 0.085 – 0.172 |
| Mt-Ka | 0.133*** | 0.082 – 0.157 |
| Pe-Nb | 0.022*** | 0.002 – 0.028 |
| Pe-Tu | 0.033*** | 0.013 – 0.036 |
| Pe-Sm | 0.036* | -0.001 – 0.048 |
| Pe-Pa | 0.114*** | 0.078 – 0.140 |
| Pe-Ps | 0.136*** | 0.088 – 0.173 |
| Pe-Ka | 0.138*** | 0.083 – 0.161 |
| Na-Nb | 0.013*** | 0.008 – 0.032 |
| Na-Sm | 0.019* | -0.005 – 0.038 |
| Na-Pa | 0.103*** | 0.073 – 0.133 |
| Na-Ps | 0.126*** | 0.081 – 0.166 |
| Na-Ka | 0.128*** | 0.078 – 0.150 |
| To-Pa | 0.071*** | 0.044 – 0.097 |
| To-Ps | 0.094*** | 0.048 – 0.132 |
| To-Ka | 0.095*** | 0.047 – 0.118 |
| Nb-Pa | 0.070*** | 0.050 – 0.091 |
| Nb-Ps | 0.094*** | 0.062 – 0.124 |
| Nb-Ka | 0.098*** | 0.061 – 0.112 |
| Tu-Pa | 0.105*** | 0.057 – 0.133 |
| Tu-Ps | 0.123*** | 0.067 – 0.156 |
| Tu-Ka | 0.126*** | 0.061 – 0.141 |
| Sm-Pa | 0.093*** | 0.053 – 0.111 |
| Sm-Ps | 0.125*** | 0.076 – 0.140 |
| Sm-Ka | 0.131*** | 0.068 – 0.136 |
