# Supplementary material for: Sea surface currents and geographic isolation shape the genetic population structure of a coral reef fish in the Indian Ocean
Source: PLoS One. 2018 Mar 9;13(3):e0193825. doi: 10.1371/journal.pone.0193825 (PMC5844546; doi:10.1371/journal.pone.0193825)
Supplement: S3 Table — (A) Pairwise ϕst-values among populations using reduced datasets. (B) Pairwise Fst-values among populations using reduced datasets. (C) Composition of reduced populations with eight randomly chosen individuals. (D) ϕct–values of hierarchical AMOVAs. (E) Fct–values of hierarchical AMOVAs. (DOCX) [file pone.0193825.s003.docx]

**S3A Table. Pairwise φ_st_-values of comparison between population of *Amphiprion akallopisos* based on sequences of mtDNA (Control Region) using one complete and two reduced datasets.** Pop comparisons: Populations that were compared. Letter codes as in Table 1. Populations composed of all available samples per population (Column 2), the first eight samples per population (Column 3), and eight randomly chosen individuals per population as mentioned in Table S3C (Column 5) as well as the differences between these values (Columns 4 (8 first – total) and 6 (8 random – total), respectively). Total: total difference between two compared datasets. P-value: significance of difference from 0 based on Z-test for comparison between a population and a fixed value. Significance level per φ_st_-value indicated: *:p<0.0241; **:p<0.01; ***: p<0.001.

| Pop comparisons | Total samples | 8 first samples | Difference Total - 8 first | 8 random samples | Difference Total - 8 random |
| --- | --- | --- | --- | --- | --- |
| La-Wa | 0.021 | 0.048 | 0.027 | 0.041 | 0.020 |
| La-Mo | 0.092 | 0.048 | -0.044 | 0.071 | -0.021 |
| La-Di | 0.092 | 0.071 | -0.021 | 0.071 | -0.021 |
| La-Ki | 0.078 | 0.143 | 0.065 | 0.086 | 0.008 |
| La-Mi | -0.030 | -0.033 | -0.003 | 0.032 | 0.062 |
| La-St | 0.059 | 0.036 | -0.023 | 0.041 | -0.018 |
| La-Dw | 0.075 | 0.082 | 0.007 | 0.048 | -0.027 |
| La-Ja | 0.060 | 0.048 | -0.012 | 0.048 | -0.012 |
| La-Ds | 0.152* | 0.114 | -0.038 | 0.095 | -0.057 |
| La-Mf | 0.065 | 0.057 | -0.008 | 0.048 | -0.017 |
| La-Mt | 0.034 | 0.048 | 0.014 | 0.041 | 0.007 |
| La-Pe | -0.006 | 0.030 | 0.036 | 0.026 | 0.032 |
| La-Na | 0.028 | 0.029 | 0.001 | 0.071 | 0.043 |
| La-To | 0.071 | 0.071 | 0.000 | 0.071 | 0.000 |
| La-Nb | 0.043 | 0.018 | -0.025 | 0.048 | 0.005 |
| La-Tu | 0.026 | 0.057 | 0.031 | 0.041 | 0.015 |
| La-Sm | 0.164* | 0.164 | 0.000 | 0.164 | 0.000 |
| La-Pa | 0.321*** | 0.321*** | 0.000 | 0.382*** | 0.061 |
| La-Ps | 0.336*** | 0.442*** | 0.106 | 0.435*** | 0.099 |
| La-Ka | 0.276*** | 0.474*** | 0.198 | 0.342*** | 0.066 |
| Wa-Mo | 0.012 | 0.000 | -0.012 | 0.000 | -0.012 |
| Wa-Di | -0.021 | 0.000 | 0.021 | 0.000 | 0.021 |
| Wa-Ki | 0.050 | 0.114 | 0.064 | 0.052 | 0.002 |
| Wa-Mi | -0.007 | 0.000 | 0.007 | 0.000 | 0.007 |
| Wa-St | 0.010 | 0.000 | -0.010 | 0.000 | -0.010 |
| Wa-Dw | -0.007 | 0.041 | 0.048 | -0.037 | -0.030 |
| Wa-Ja | -0.010 | 0.000 | 0.010 | 0.000 | 0.010 |
| Wa-Ds | 0.017* | 0.057 | 0.040 | 0.000 | -0.017 |
| Wa-Mf | -0.002 | 0.000 | 0.002 | 0.000 | 0.002 |
| Wa-Mt | 0.009 | 0.000 | -0.009 | 0.000 | -0.009 |
| Wa-Pe | 0.004 | 0.015 | 0.011 | 0.000 | -0.004 |
| Wa-Na | -0.005 | 0.000 | 0.005 | 0.000 | 0.005 |
| Wa-To | -0.035 | 0.000 | 0.035 | 0.000 | 0.035 |
| Wa-Nb | 0.005 | -0.016 | -0.021 | 0.000 | -0.005 |
| Wa-Tu | 0.000 | 0.000 | 0.000 | 0.000 | 0.000 |
| Wa-Sm | 0.191*** | 0.150 | -0.041 | 0.143 | -0.048 |
| Wa-Pa | 0.391*** | 0.301*** | -0.090 | 0.351*** | -0.040 |
| Wa-Ps | 0.393*** | 0.433*** | 0.040 | 0.393*** | 0.000 |
| Wa-Ka | 0.342*** | 0.458*** | 0.116 | 0.305*** | -0.037 |
| Mo-Di | -0.009 | -0.067 | -0.058 | 0.000 | 0.009 |
| Mo-Ki | 0.096* | 0.114 | 0.018 | 0.071 | -0.025 |
| Mo-Mi | 0.014 | 0.000 | -0.014 | 0.000 | -0.014 |
| Mo-St | 0.009 | -0.032 | -0.041 | -0.053 | -0.062 |
| Mo-Dw | 0.014 | 0.005 | -0.009 | -0.067 | -0.081 |
| Mo-Ja | -0.003 | -0.043 | -0.040 | -0.067 | -0.064 |
| Mo-Ds | 0.039 | 0.057 | 0.018 | 0.000 | -0.039 |
| Mo-Mf | 0.015 | 0.000 | -0.015 | 0.000 | -0.015 |
| Mo-Mt | -0.000 | -0.043 | -0.043 | 0.000 | 0.000 |
| Mo-Pe | -0.003 | 0.002 | 0.005 | 0.000 | 0.003 |
| Mo-Na | -0.009 | 0.000 | 0.009 | 0.000 | 0.009 |
| Mo-To | -0.079 | -0.067 | 0.012 | 0.000 | 0.079 |
| Mo-Nb | -0.011 | -0.016 | -0.005 | 0.000 | 0.011 |
| Mo-Tu | 0.009 | 0.000 | -0.009 | 0.000 | -0.009 |
| Mo-Sm | 0.271*** | 0.150 | -0.121 | 0.167 | -0.104 |
| Mo-Pa | 0.425*** | 0.288*** | -0.137 | 0.382*** | -0.043 |
| Mo-Ps | 0.435*** | 0.421*** | -0.014 | 0.440*** | 0.005 |
| Mo-Ka | 0.384*** | 0.455*** | 0.071 | 0.348*** | -0.036 |
| Di-Ki | 0.079* | 0.143 | 0.064 | 0.071 | -0.008 |
| Di-Mi | -0.006 | 0.000 | 0.006 | 0.000 | 0.006 |
| Di-St | -0.001 | 0.000 | 0.001 | 0.000 | 0.001 |
| Di-Dw | 0.009 | 0.057 | 0.048 | 0.000 | -0.009 |
| Di-Ja | 0.004 | 0.000 | -0.004 | 0.000 | -0.004 |
| Di-Ds | 0.029 | 0.095 | 0.066 | 0.000 | -0.029 |
| Di-Mf | -0.003 | 0.000 | 0.003 | 0.000 | 0.003 |
| Di-Mt | -0.010 | 0.000 | 0.010 | 0.000 | 0.010 |
| Di-Pe | -0.012 | 0.017 | 0.029 | 0.000 | 0.012 |
| Di-Na | -0.019 | 0.000 | 0.019 | 0.000 | 0.019 |
| Di-To | -0.005 | 0.000 | 0.005 | 0.000 | 0.005 |
| Di-Nb | -0.002 | 0.000 | 0.002 | 0.000 | 0.002 |
| Di-Tu | -0.011 | 0.000 | 0.011 | 0.000 | 0.011 |
| Di-Sm | 0.235*** | 0.167 | -0.068 | 0.167 | -0.068 |
| Di-Pa | 0.394*** | 0.328*** | -0.066 | 0.389*** | -0.005 |
| Di-Ps | 0.408*** | 0.450*** | 0.042 | 0.444*** | 0.036 |
| Di-Ka | 0.355*** | 0.482*** | 0.127 | 0.348*** | -0.007 |
| Ki-Mi | 0.040 | 0.104 | 0.064 | 0.025 | -0.015 |
| Ki-St | 0.065 | 0.056 | -0.009 | 0.030 | -0.035 |
| Ki-Dw | 0.087*** | 0.130 | 0.043 | 0.057 | -0.030 |
| Ki-Ja | 0.038 | 0.114 | 0.076 | 0.033 | -0.005 |
| Ki-Ds | 0.124*** | 0.159 | 0.035 | 0.082 | -0.042 |
| Ki-Mf | 0.026 | 0.048 | 0.022 | -0.019 | -0.045 |
| Ki-Mt | 0.064** | 0.114 | 0.050 | 0.030 | -0.034 |
| Ki-Pe | 0.021 | 0.031 | 0.010 | 0.005 | -0.016 |
| Ki-Na | 0.060** | 0.082 | 0.022 | 0.071 | 0.011 |
| Ki-To | 0.045 | 0.143 | 0.098 | 0.071 | 0.026 |
| Ki-Nb | 0.072*** | 0.057 | -0.015 | 0.057 | -0.015 |
| Ki-Tu | 0.056* | 0.127 | 0.071 | 0.052 | -0.004 |
| Ki-Sm | 0.188*** | 0.173* | -0.015 | 0.149 | -0.039 |
| Ki-Pa | 0.3551*** | 0.306*** | -0.049 | 0.351*** | -0.004 |
| Ki-Ps | 0.366*** | 0.419*** | 0.053 | 0.400*** | 0.034 |
| Ki-Ka | 0.313*** | 0.452*** | 0.139 | 0.313*** | 0.000 |
| Mi-St | 0.011 | 0.000 | -0.011 | 0.000 | -0.011 |
| Mi-Dw | 0.003 | 0.036 | 0.033 | 0.000 | -0.003 |
| Mi-Ja | -0.022 | -0.037 | -0.015 | -0.029 | -0.007 |
| Mi-Ds | 0.020 | 0.048 | 0.028 | 0.000 | -0.020 |
| Mi-Mf | 0.000 | 0.000 | 0.000 | 0.000 | 0.000 |
| Mi-Mt | 0.006 | 0.000 | -0.006 | -0.026 | -0.032 |
| Mi-Pe | 0.001 | 0.014 | 0.013 | 0.000 | -0.001 |
| Mi-Na | 0.005 | 0.000 | -0.005 | 0.000 | -0.005 |
| Mi-To | -0.034 | 0.000 | 0.034 | 0.000 | 0.034 |
| Mi-Nb | -0.005 | -0.030 | -0.025 | 0.000 | 0.005 |
| Mi-Tu | 0.001 | 0.000 | -0.001 | 0.000 | -0.001 |
| Mi-Sm | 0.182*** | 0.138 | -0.044 | 0.126 | -0.056 |
| Mi-Pa | 0.389*** | 0.311*** | -0.078 | 0.360*** | -0.029 |
| Mi-Ps | 0.397*** | 0.425*** | 0.028 | 0.399*** | 0.002 |
| Mi-Ka | 0.342*** | 0.457*** | 0.115 | 0.311*** | -0.031 |
| St-Dw | 0.009 | 0.004 | -0.005 | -0.037 | -0.046 |
| St-Ja | -0.018 | -0.032 | -0.014 | -0.037 | -0.019 |
| St-Ds | 0.021 | 0.041 | 0.020 | 0.000 | -0.021 |
| St-Mf | -0.005 | 0.000 | 0.005 | -0.037 | -0.032 |
| St-Mt | 0.016 | 0.000 | -0.016 | 0.000 | -0.016 |
| St-Pe | 0.008 | 0.014 | 0.006 | 0.000 | -0.008 |
| St-Na | -0.000 | 0.000 | 0.000 | 0.000 | 0.000 |
| St-To | -0.021 | 0.000 | 0.021 | 0.000 | 0.021 |
| St-Nb | 0.016 | 0.000 | -0.016 | 0.000 | -0.016 |
| St-Tu | 0.008 | 0.000 | -0.008 | 0.000 | -0.008 |
| St-Sm | 0.262*** | 0.136 | -0.126 | 0.143 | -0.119 |
| St-Pa | 0.455*** | 0.290*** | -0.165 | 0.361*** | -0.094 |
| St-Ps | 0.468*** | 0.419*** | -0.049 | 0.420*** | -0.048 |
| St-Ka | 0.420*** | 0.452*** | 0.032 | 0.330*** | -0.090 |
| Dw-Ja | -0.007 | 0.005 | 0.012 | -0.043 | -0.036 |
| Dw-Ds | 0.034 | 0.095 | 0.061 | 0.000 | -0.034 |
| Dw-Mf | 0.012 | 0.048 | 0.036 | 0.000 | -0.012 |
| Dw-Mt | 0.012 | 0.041 | 0.029 | 0.000 | -0.012 |
| Dw-Pe | 0.006 | 0.029 | 0.023 | 0.000 | -0.006 |
| Dw-Na | -0.006 | 0.026 | 0.032 | 0.000 | 0.006 |
| Dw-To | -0.009 | 0.057 | 0.066 | 0.000 | 0.009 |
| Dw-Nb | 0.004 | -0.013 | -0.017 | 0.000 | -0.004 |
| Dw-Tu | 0.008 | 0.048 | 0.040 | 0.000 | -0.008 |
| Dw-Sm | 0.258*** | 0.156 | -0.102 | 0.150 | -0.108 |
| Dw-Pa | 0.429*** | 0.284*** | -0.145 | 0.364*** | -0.065 |
| Dw-Ps | 0.435*** | 0.407*** | -0.028 | 0.411*** | -0.024 |
| Dw-Ka | 0.391*** | 0.443*** | 0.052 | 0.325*** | -0.066 |
| Ja-Ds | 0.039 | 0.057 | 0.018 | 0.000 | -0.039 |
| Ja-Mf | -0.011 | 0.000 | 0.011 | 0.000 | 0.011 |
| Ja-Mt | -0.018 | 0.000 | 0.018 | -0.037 | -0.019 |
| Ja-Pe | -0.016 | 0.015 | 0.031 | 0.000 | 0.016 |
| Ja-Na | -0.013 | 0.000 | 0.013 | 0.000 | 0.013 |
| Ja-To | -0.013 | 0.000 | 0.013 | 0.000 | 0.013 |
| Ja-Nb | 0.000 | -0.016 | -0.016 | 0.000 | 0.000 |
| Ja-Tu | -0.010 | 0.000 | 0.010 | 0.000 | 0.010 |
| Ja-Sm | 0.196* | 0.150 | -0.046 | 0.150 | -0.046 |
| Ja-Pa | 0.355*** | 0.310*** | -0.045 | 0.370*** | 0.015 |
| Ja-Ps | 0.369*** | 0.432*** | 0.063 | 0.420*** | 0.051 |
| Ja-Ka | 0.318*** | 0.464*** | 0.146 | 0.333*** | 0.015 |
| Ds-Mf | 0.023 | 0.071 | 0.048 | 0.000 | -0.023 |
| Ds-Mt | 0.012 | 0.057 | 0.045 | 0.000 | -0.012 |
| Ds-Pe | 0.005 | 0.032 | 0.027 | 0.000 | -0.005 |
| Ds-Na | -0.002 | 0.032 | 0.034 | 0.000 | 0.002 |
| Ds-To | 0.035 | 0.095 | 0.060 | 0.000 | -0.035 |
| Ds-Nb | 0.019 | 0.019 | 0.000 | 0.000 | -0.019 |
| Ds-Tu | 0.012 | 0.071 | 0.059 | 0.000 | -0.012 |
| Ds-Sm | 0.310** | 0.173* | -0.137 | 0.176* | -0.134 |
| Ds-Pa | 0.461*** | 0.327*** | -0.134 | 0.394*** | -0.067 |
| Ds-Ps | 0.475*** | 0.448*** | -0.027 | 0.451*** | -0.024 |
| Ds-Ka | 0.428*** | 0.480*** | 0.052 | 0.353*** | -0.075 |
| Mf-Mt | 0.005 | 0.000 | -0.005 | 0.000 | -0.005 |
| Mf-Pe | -0.008 | 0.002 | 0.010 | -0.023 | -0.015 |
| Mf-Na | -0.008 | 0.000 | 0.008 | 0.000 | 0.008 |
| Mf-To | -0.020 | 0.000 | 0.020 | 0.000 | 0.020 |
| Mf-Nb | 0.006 | 0.000 | -0.006 | 0.000 | -0.006 |
| Mf-Tu | -0.003 | 0.000 | 0.003 | 0.000 | 0.003 |
| Mf-Sm | 0.234** | 0.158 | -0.076 | 0.150 | -0.084 |
| Mf-Pa | 0.405*** | 0.322*** | -0.083 | 0.368*** | -0.037 |
| Mf-Ps | 0.421*** | 0.442*** | 0.021 | 0.426*** | 0.005 |
| Mf-Ka | 0.369*** | 0.475*** | 0.106 | 0.333*** | -0.036 |
| Mt-Pe | 0.016* | 0.002 | -0.014 | 0.000 | -0.016 |
| Mt-Na | 0.007 | 0.000 | -0.007 | 0.000 | -0.007 |
| Mt-To | -0.045 | -0.067 | -0.022 | 0.000 | 0.045 |
| Mt-Nb | 0.011 | -0.016 | -0.027 | 0.000 | -0.011 |
| Mt-Tu | 0.002 | 0.000 | -0.002 | -0.032 | -0.034 |
| Mt-Sm | 0.268*** | 0.150 | -0.118 | 0.143 | -0.125 |
| Mt-Pa | 0.522*** | 0.293*** | -0.229 | 0.372*** | -0.150 |
| Mt-Ps | 0.522*** | 0.420*** | -0.102 | 0.418*** | -0.104 |
| Mt-Ka | 0.473*** | 0.456*** | -0.017 | 0.328*** | -0.145 |
| Pe-Na | 0.006 | 0.002 | -0.004 | 0.000 | -0.006 |
| Pe-To | -0.047 | 0.002 | 0.049 | 0.000 | 0.047 |
| Pe-Nb | -0.002 | -0.003 | -0.001 | -0.011 | -0.009 |
| Pe-Tu | -0.008 | 0.002 | 0.010 | -0.011 | -0.003 |
| Pe-Sm | 0.117*** | 0.064 | -0.053 | 0.097 | -0.020 |
| Pe-Pa | 0.454*** | 0.236*** | -0.218 | 0.348*** | -0.106 |
| Pe-Ps | 0.446*** | 0.355*** | -0.091 | 0.393*** | -0.053 |
| Pe-Ka | 0.392*** | 0.395*** | 0.003 | 0.310*** | -0.082 |
| Na-To | -0.053 | 0.000 | 0.053 | -0.143 | -0.090 |
| Na-Nb | 0.001 | 0.000 | -0.001 | 0.000 | -0.001 |
| Na-Tu | -0.001 | 0.000 | 0.001 | 0.000 | 0.001 |
| Na-Sm | 0.258*** | 0.125 | -0.133 | 0.167 | -0.091 |
| Na-Pa | 0.501*** | 0.295*** | -0.206 | 0.327*** | -0.174 |
| Na-Ps | 0.504*** | 0.414*** | -0.090 | 0.426*** | -0.078 |
| Na-Ka | 0.457*** | 0.449*** | -0.008 | 0.372*** | -0.085 |
| To-Nb | -0.059 | 0.000 | 0.059 | 0.000 | 0.059 |
| To-Tu | -0.034 | -0.018 | 0.016 | 0.000 | 0.034 |
| To-Sm | 0.167 | 0.167 | 0.000 | 0.167 | 0.000 |
| To-Pa | 0.306*** | 0.306*** | 0.000 | 0.327*** | 0.021 |
| To-Ps | 0.318*** | 0.435*** | 0.117 | 0.426*** | 0.108 |
| To-Ka | 0.254*** | 0.469*** | 0.215 | 0.372*** | 0.118 |
| Nb-Tu | -0.001 | 0.000 | 0.001 | -0.077 | -0.076 |
| Nb-Sm | 0.217** | 0.069 | -0.148 | 0.062 | -0.155 |
| Nb-Pa | 0.453*** | 0.200*** | -0.253 | 0.374*** | -0.079 |
| Nb-Ps | 0.457*** | 0.296*** | -0.161 | 0.426*** | -0.031 |
| Nb-Ka | 0.406*** | 0.331*** | -0.075 | 0.333*** | -0.073 |
| Tu-Sm | 0.173*** | 0.158 | -0.015 | 0.059 | -0.114 |
| Tu-Pa | 0.423*** | 0.319*** | -0.104 | 0.370*** | -0.053 |
| Tu-Ps | 0.431*** | 0.440*** | 0.009 | 0.420*** | -0.011 |
| Tu-Ka | 0.379*** | 0.476*** | 0.097 | 0.328*** | -0.051 |
| Sm-Pa | 0.302*** | 0.284*** | -0.018 | 0.336*** | 0.034 |
| Sm-Ps | 0.307*** | 0.387*** | 0.080 | 0.377*** | 0.070 |
| Sm-Ka | 0.250*** | 0.425*** | 0.175 | 0.297*** | 0.047 |
| Pa-Ps | 0.009 | -0.073 | -0.082 | -0.087 | -0.096 |
| Pa-Ka | -0.045 | -0.003 | 0.042 | 0.012 | 0.057 |
| Ps-Ka | 0.008 | 0.040 | 0.032 | -0.020 | -0.028 |
|  |  | Total | -0.145 (p=0.57) |  | -3.537 (p=0.99) |

**S3B Table. Pairwise F_st_-values of comparison between population of *Amphiprion akallopisos* based on microsatellite data from 13 loci (mentioned in Table 2) using one complete and two reduced datasets.** Pop comparisons: Populations that were compared. Letter codes as in Table 1. Populations composed of all available samples per population (Column 2), the first eight samples per population (Column 3), and eight randomly chosen individuals per population as mentioned in Table S3C (Column 5) as well as the differences between these values (Columns 4 (8 first – total) and 6 (8 random – total) respectively). Total: total difference between two compared datasets. P-value: significance of difference from 0 based on Z-test for comparison between a population and a fixed value. Significance level per φ_st_-value indicated: *:p<0.0241; **:p<0.01; ***: p<0.001.

| Pop comparisons | Total samples | 8 first samples | Difference Total - 8 first | 8 random samples | Difference Total - 8 random |
| --- | --- | --- | --- | --- | --- |
| La-Wa | -0.001 | -0.001 | 0 | -0.027 | -0.026 |
| La-Mo | 0.011 | 0.011 | 0 | 0.002 | -0.009 |
| La-Di | 0.017 | -0.004 | -0.021 | -0.003 | -0.02 |
| La-Ki | 0.022 | 0.002 | -0.02 | -0.011 | -0.033 |
| La-Mi | 0.015 | -0.002 | -0.017 | -0.012 | -0.027 |
| La-St | 0.013 | -0.017 | -0.03 | -0.030 | -0.043 |
| La-Dw | 0.007 | -0.020 | -0.027 | -0.024 | -0.031 |
| La-Ja | 0.025 | 0.024 | -0.001 | -0.006 | -0.031 |
| La-Ds | 0.022 | -0.011 | -0.033 | 0.016 | -0.006 |
| La-Mf | 0.026* | 0.003 | -0.023 | -0.009 | -0.035 |
| La-Mt | 0.008 | -0.009 | -0.017 | -0.018 | -0.026 |
| La-Pe | 0.021* | -0.006 | -0.027 | 0.017 | -0.004 |
| La-Na | 0.012 | -0.019 | -0.031 | -0.007 | -0.019 |
| La-To | -0.010 | -0.017 | -0.007 | -0.017 | -0.007 |
| La-Nb | 0.014 | 0.035 | 0.021 | 0.015 | 0.001 |
| La-Tu | 0.024* | 0.028 | 0.004 | 0.004 | -0.02 |
| La-Sm | 0.017 | 0.015 | -0.002 | 0.015 | -0.002 |
| La-Pa | 0.077*** | 0.092*** | 0.015 | 0.097*** | 0.02 |
| La-Ps | 0.101*** | 0.121*** | 0.02 | 0.133*** | 0.032 |
| La-Ka | 0.112*** | 0.139*** | 0.027 | 0.161*** | 0.049 |
| Wa-Mo | 0.031* | 0.028 | -0.003 | -0.005 | -0.036 |
| Wa-Di | 0.038*** | 0.006 | -0.032 | -0.012 | -0.05 |
| Wa-Ki | 0.049*** | 0.015 | -0.034 | 0.015 | -0.034 |
| Wa-Mi | 0.020 | -0.008 | -0.028 | -0.010 | -0.03 |
| Wa-St | 0.029** | -0.006 | -0.035 | -0.021 | -0.05 |
| Wa-Dw | 0.031*** | -0.006 | -0.037 | -0.020 | -0.051 |
| Wa-Ja | 0.040*** | 0.015 | -0.025 | -0.009 | -0.049 |
| Wa-Ds | 0.044*** | 0.006 | -0.038 | 0.003 | -0.041 |
| Wa-Mf | 0.059*** | 0.011 | -0.048 | 0.013 | -0.046 |
| Wa-Mt | 0.022** | -0.004 | -0.026 | -0.019 | -0.041 |
| Wa-Pe | 0.026*** | -0.020 | -0.046 | 0.018 | -0.008 |
| Wa-Na | 0.031*** | -0.009 | -0.04 | -0.008 | -0.039 |
| Wa-To | 0.021 | -0.002 | -0.023 | -0.011 | -0.032 |
| Wa-Nb | 0.037*** | 0.042** | 0.005 | 0.029 | -0.008 |
| Wa-Tu | 0.049*** | 0.059** | 0.01 | -0.004 | -0.053 |
| Wa-Sm | 0.028 | -0.011 | -0.039 | 0.011 | -0.017 |
| Wa-Pa | 0.080*** | 0.071*** | -0.009 | 0.080** | 0 |
| Wa-Ps | 0.108*** | 0.111*** | 0.003 | 0.123*** | 0.015 |
| Wa-Ka | 0.113*** | 0.118*** | 0.005 | 0.150*** | 0.037 |
| Mo-Di | -0.004 | 0.008 | 0.012 | -0.012 | -0.008 |
| Mo-Ki | 0.012 | 0.001 | -0.011 | 0.027 | 0.015 |
| Mo-Mi | 0.011 | 0.036* | 0.025 | 0.012 | 0.001 |
| Mo-St | -0.005 | 0.000 | 0.005 | -0.030 | -0.025 |
| Mo-Dw | 0.001 | 0.018 | 0.017 | -0.001 | -0.002 |
| Mo-Ja | 0.021 | 0.020 | -0.001 | 0.005 | -0.016 |
| Mo-Ds | -0.001 | -0.016 | -0.015 | 0.018 | 0.019 |
| Mo-Mf | 0.014 | 0.027 | 0.013 | 0.023 | 0.009 |
| Mo-Mt | 0.005 | 0.018 | 0.013 | -0.004 | -0.009 |
| Mo-Pe | 0.003 | 0.007 | 0.004 | -0.014 | -0.017 |
| Mo-Na | -0.002 | -0.023 | -0.021 | -0.018 | -0.016 |
| Mo-To | 0.004 | 0.000 | -0.004 | 0.010 | 0.006 |
| Mo-Nb | 0.020* | 0.053** | 0.033 | 0.021 | 0.001 |
| Mo-Tu | 0.026*** | 0.072* | 0.046 | 0.026 | 0 |
| Mo-Sm | 0.024 | 0.044 | 0.02 | 0.025 | 0.001 |
| Mo-Pa | 0.098*** | 0.112*** | 0.014 | 0.058** | -0.04 |
| Mo-Ps | 0.118*** | 0.129*** | 0.011 | 0.096** | -0.022 |
| Mo-Ka | 0.126*** | 0.146*** | 0.02 | 0.128*** | 0.002 |
| Di-Ki | 0.006 | 0.022 | 0.016 | 0.019 | 0.013 |
| Di-Mi | 0.019* | 0.013 | -0.006 | 0.020 | 0.001 |
| Di-St | -0.007 | -0.019 | -0.012 | -0.015 | -0.008 |
| Di-Dw | -0.008 | -0.015 | -0.007 | -0.005 | 0.003 |
| Di-Ja | 0.009 | 0.018 | 0.009 | -0.008 | -0.017 |
| Di-Ds | -0.008 | -0.016 | -0.008 | -0.005 | 0.003 |
| Di-Mf | 0.001 | -0.002 | -0.003 | 0.015 | 0.014 |
| Di-Mt | 0.005 | -0.012 | -0.017 | 0.008 | 0.003 |
| Di-Pe | 0.004 | 0.006 | 0.002 | 0.004 | 0 |
| Di-Na | 0.000 | -0.002 | -0.002 | -0.014 | -0.014 |
| Di-To | -0.007 | -0.009 | -0.002 | 0.003 | 0.01 |
| Di-Nb | 0.031*** | 0.040* | 0.009 | 0.059** | 0.028 |
| Di-Tu | 0.032*** | 0.070*** | 0.038 | 0.025 | -0.007 |
| Di-Sm | 0.031* | 0.033 | 0.002 | 0.017 | -0.014 |
| Di-Pa | 0.098*** | 0.083*** | -0.015 | 0.089*** | -0.009 |
| Di-Ps | 0.124*** | 0.126*** | 0.002 | 0.133*** | 0.009 |
| Di-Ka | 0.124*** | 0.136*** | 0.012 | 0.149*** | 0.025 |
| Ki-Mi | 0.006 | 0.001 | -0.005 | 0.005 | -0.001 |
| Ki-St | 0.007 | 0.009 | 0.002 | -0.001 | -0.008 |
| Ki-Dw | 0.008 | 0.000 | -0.008 | 0.004 | -0.004 |
| Ki-Ja | 0.015 | 0.050 | 0.035 | 0.002 | -0.013 |
| Ki-Ds | 0.021* | 0.013 | -0.008 | 0.053*** | 0.032 |
| Ki-Mf | 0.000 | 0.025 | 0.025 | -0.002 | -0.002 |
| Ki-Mt | 0.008 | 0.006 | -0.002 | 0.012 | 0.004 |
| Ki-Pe | 0.011* | 0.008 | -0.003 | 0.015 | 0.004 |
| Ki-Na | 0.004 | -0.008 | -0.012 | 0.002 | -0.002 |
| Ki-To | 0.003 | 0.001 | -0.002 | 0.008 | 0.005 |
| Ki-Nb | 0.031*** | 0.059** | 0.028 | 0.031 | 0 |
| Ki-Tu | 0.041*** | 0.075*** | 0.034 | 0.045* | 0.004 |
| Ki-Sm | 0.028* | 0.030 | 0.002 | 0.027 | -0.001 |
| Ki-Pa | 0.122*** | 0.109*** | -0.013 | 0.121*** | -0.001 |
| Ki-Ps | 0.143*** | 0.138*** | -0.005 | 0.157*** | 0.014 |
| Ki-Ka | 0.151*** | 0.159*** | 0.008 | 0.172*** | 0.021 |
| Mi-St | 0.002 | 0.006 | 0.004 | -0.025 | -0.027 |
| Mi-Dw | 0.002 | -0.024 | -0.026 | -0.035 | -0.037 |
| Mi-Ja | 0.018* | 0.039 | 0.021 | 0.002 | -0.016 |
| Mi-Ds | 0.009 | 0.004 | -0.005 | 0.008 | -0.001 |
| Mi-Mf | 0.033*** | 0.040 | 0.007 | 0.027 | -0.006 |
| Mi-Mt | -0.001 | 0.012 | 0.013 | -0.021 | -0.02 |
| Mi-Pe | -0.004 | 0.001 | 0.005 | 0.006 | 0.01 |
| Mi-Na | 0.004 | 0.010 | 0.006 | 0.016 | 0.012 |
| Mi-To | 0.007 | -0.003 | -0.01 | -0.001 | -0.008 |
| Mi-Nb | 0.018* | 0.014 | -0.004 | 0.026 | 0.008 |
| Mi-Tu | 0.021* | 0.056** | 0.035 | 0.006 | -0.015 |
| Mi-Sm | 0.006 | 0.004 | -0.002 | -0.007 | -0.013 |
| Mi-Pa | 0.116*** | 0.079*** | -0.037 | 0.097*** | -0.019 |
| Mi-Ps | 0.141*** | 0.128*** | -0.013 | 0.128*** | -0.013 |
| Mi-Ka | 0.147*** | 0.140*** | -0.007 | 0.162** | 0.015 |
| St-Dw | -0.009 | 0.008 | 0.017 | -0.029 | -0.02 |
| St-Ja | 0.007 | 0.037* | 0.03 | -0.018 | -0.025 |
| St-Ds | -0.010 | -0.021 | -0.011 | -0.004 | 0.006 |
| St-Mf | 0.015* | 0.029 | 0.014 | 0.014 | -0.001 |
| St-Mt | -0.003 | -0.042 | -0.039 | -0.026 | -0.023 |
| St-Pe | -0.006 | -0.012 | -0.006 | -0.027 | -0.021 |
| St-Na | -0.006 | -0.012 | -0.006 | -0.022 | -0.016 |
| St-To | -0.013 | -0.016 | -0.003 | -0.033 | -0.02 |
| St-Nb | 0.019* | 0.036 | 0.017 | 0.003 | -0.016 |
| St-Tu | 0.014* | 0.059* | 0.045 | 0.013 | -0.001 |
| St-Sm | 0.045* | 0.038* | -0.007 | 0.005 | -0.04 |
| St-Pa | 0.097*** | 0.092** | -0.005 | 0.055*** | -0.042 |
| St-Ps | 0.122*** | 0.136*** | 0.014 | 0.101*** | -0.021 |
| St-Ka | 0.122*** | 0.142*** | 0.02 | 0.129*** | 0.007 |
| Dw-Ja | -0.001 | -0.005 | -0.004 | -0.019 | -0.018 |
| Dw-Ds | -0.003 | -0.011 | -0.008 | 0.007 | 0.01 |
| Dw-Mf | 0.009 | -0.002 | -0.011 | 0.008 | -0.001 |
| Dw-Mt | -0.007 | 0.005 | 0.012 | -0.017 | -0.01 |
| Dw-Pe | -0.003 | -0.006 | -0.003 | 0.013 | 0.016 |
| Dw-Na | -0.003 | -0.005 | -0.002 | -0.000 | 0.003 |
| Dw-To | -0.019 | -0.029 | -0.01 | -0.017 | 0.002 |
| Dw-Nb | 0.009 | 0.011 | 0.002 | 0.020 | 0.011 |
| Dw-Tu | 0.018* | 0.034* | 0.016 | 0.001 | -0.017 |
| Dw-Sm | 0.018 | 0.003 | -0.015 | -0.018 | -0.036 |
| Dw-Pa | 0.096*** | 0.057* | -0.039 | 0.086*** | -0.01 |
| Dw-Ps | 0.115*** | 0.101*** | -0.014 | 0.118*** | 0.003 |
| Dw-Ka | 0.117*** | 0.121*** | 0.004 | 0.145*** | 0.028 |
| Ja-Ds | 0.012 | 0.025 | 0.013 | -0.004 | -0.016 |
| Ja-Mf | -0.006 | -0.004 | 0.002 | -0.020 | -0.014 |
| Ja-Mt | 0.016* | 0.047*** | 0.031 | -0.000 | -0.016 |
| Ja-Pe | 0.018* | 0.015 | -0.003 | 0.017 | -0.001 |
| Ja-Na | 0.009 | 0.024 | 0.015 | -0.005 | -0.014 |
| Ja-To | -0.007 | 0.004 | 0.011 | -0.027 | -0.02 |
| Ja-Nb | 0.015 | 0.057 | 0.042 | 0.026 | 0.011 |
| Ja-Tu | 0.017 | 0.057 | 0.04 | -0.009 | -0.026 |
| Ja-Sm | 0.026 | 0.055* | 0.029 | 0.006 | -0.02 |
| Ja-Pa | 0.101*** | 0.092*** | -0.009 | 0.083*** | -0.018 |
| Ja-Ps | 0.120*** | 0.125*** | 0.005 | 0.121*** | 0.001 |
| Ja-Ka | 0.118*** | 0.125*** | 0.007 | 0.125*** | 0.007 |
| Ds-Mf | 0.015* | 0.036** | 0.021 | 0.029 | 0.014 |
| Ds-Mt | 0.008* | -0.014 | -0.022 | 0.015 | 0.007 |
| Ds-Pe | 0.004 | -0.002 | -0.006 | 0.016 | 0.012 |
| Ds-Na | -0.000 | -0.005 | -0.005 | 0.007 | 0.007 |
| Ds-To | -0.002 | -0.020 | -0.018 | 0.007 | 0.009 |
| Ds-Nb | 0.030*** | 0.022** | -0.008 | 0.077* | 0.047 |
| Ds-Tu | 0.040*** | 0.071*** | 0.031 | 0.010 | -0.03 |
| Ds-Sm | 0.011* | 0.035** | 0.024 | 0.037 | 0.026 |
| Ds-Pa | 0.118*** | 0.088*** | -0.03 | 0.108*** | -0.01 |
| Ds-Ps | 0.137*** | 0.128*** | -0.009 | 0.144*** | 0.007 |
| Ds-Ka | 0.141*** | 0.136*** | -0.005 | 0.164*** | 0.023 |
| Mf-Mt | 0.018** | 0.052*** | 0.034 | 0.010 | -0.008 |
| Mf-Pe | 0.024*** | 0.019 | -0.005 | 0.034 | 0.01 |
| Mf-Na | 0.009 | -0.005 | -0.014 | -0.006 | -0.015 |
| Mf-To | 0.001 | 0.008 | 0.007 | 0.004 | 0.003 |
| Mf-Nb | 0.022*** | 0.069** | 0.047 | 0.025* | 0.003 |
| Mf-Tu | 0.033*** | 0.066*** | 0.033 | 0.017 | -0.016 |
| Mf-Sm | 0.036* | 0.040 | 0.004 | 0.037* | 0.001 |
| Mf-Pa | 0.114*** | 0.097*** | -0.017 | 0.100*** | -0.014 |
| Mf-Ps | 0.136*** | 0.134*** | -0.002 | 0.140*** | 0.004 |
| Mf-Ka | 0.138*** | 0.136*** | -0.002 | 0.146*** | 0.008 |
| Mt-Pe | -0.002 | -0.005 | -0.003 | 0.007 | 0.009 |
| Mt-Na | -0.003 | 0.001 | 0.004 | -0.015 | -0.012 |
| Mt-To | -0.008 | -0.015 | -0.007 | -0.015 | -0.007 |
| Mt-Nb | 0.016*** | 0.019 | 0.003 | 0.023 | 0.007 |
| Mt-Tu | 0.019** | 0.036 | 0.017 | 0.022 | 0.003 |
| Mt-Sm | 0.013 | 0.020 | 0.007 | 0.023** | 0.01 |
| Mt-Pa | 0.105*** | 0.060*** | -0.045 | 0.096*** | -0.009 |
| Mt-Ps | 0.133*** | 0.093*** | -0.04 | 0.129*** | -0.004 |
| Mt-Ka | 0.133*** | 0.109*** | -0.024 | 0.155*** | 0.022 |
| Pe-Na | -0.003 | -0.017 | -0.014 | -0.002 | 0.001 |
| Pe-To | 0.001 | -0.009 | -0.01 | 0.026 | 0.025 |
| Pe-Nb | 0.022*** | 0.029 | 0.007 | 0.050** | 0.028 |
| Pe-Tu | 0.033*** | 0.038 | 0.005 | 0.067** | 0.034 |
| Pe-Sm | 0.036* | 0.015 | -0.021 | 0.056*** | 0.02 |
| Pe-Pa | 0.114*** | 0.078*** | -0.036 | 0.104*** | -0.01 |
| Pe-Ps | 0.136*** | 0.108*** | -0.028 | 0.140*** | 0.004 |
| Pe-Ka | 0.138*** | 0.125*** | -0.013 | 0.181*** | 0.043 |
| Na-To | -0.009 | -0.005 | 0.004 | -0.013 | -0.004 |
| Na-Nb | 0.013*** | 0.018 | 0.005 | 0.013 | 0 |
| Na-Tu | 0.007 | 0.030 | 0.023 | 0.013 | 0.006 |
| Na-Sm | 0.019* | -0.005 | -0.024 | 0.029 | 0.01 |
| Na-Pa | 0.103*** | 0.073** | -0.03 | 0.071*** | -0.032 |
| Na-Ps | 0.126*** | 0.107*** | -0.019 | 0.102*** | -0.024 |
| Na-Ka | 0.128*** | 0.123*** | -0.005 | 0.116*** | -0.012 |
| To-Nb | -0.006 | 0.014 | 0.02 | 0.003 | 0.009 |
| To-Tu | 0.001 | 0.015 | 0.014 | -0.000 | -0.001 |
| To-Sm | 0.002 | 0.002 | 0 | 0.002 | 0 |
| To-Pa | 0.071*** | 0.055*** | -0.016 | 0.066*** | -0.005 |
| To-Ps | 0.094*** | 0.089*** | -0.005 | 0.100*** | 0.006 |
| To-Ka | 0.095*** | 0.101*** | 0.006 | 0.116*** | 0.021 |
| Nb-Tu | 0.012 | 0.033 | 0.021 | 0.021 | 0.009 |
| Nb-Sm | 0.001 | 0.028 | 0.027 | 0.013 | 0.012 |
| Nb-Pa | 0.070*** | 0.028 | -0.042 | 0.048** | -0.022 |
| Nb-Ps | 0.094*** | 0.070*** | -0.024 | 0.096*** | 0.002 |
| Nb-Ka | 0.098*** | 0.081*** | -0.017 | 0.116*** | 0.018 |
| Tu-Sm | 0.025 | 0.037 | 0.012 | 0.001 | -0.024 |
| Tu-Pa | 0.105*** | 0.094*** | -0.011 | 0.066*** | -0.039 |
| Tu-Ps | 0.123*** | 0.118*** | -0.005 | 0.106*** | -0.017 |
| Tu-Ka | 0.126*** | 0.111*** | -0.015 | 0.120*** | -0.006 |
| Sm-Pa | 0.093*** | 0.076*** | -0.017 | 0.092*** | -0.001 |
| Sm-Ps | 0.125*** | 0.121*** | -0.004 | 0.133*** | 0.008 |
| Sm-Ka | 0.131*** | 0.139*** | 0.008 | 0.158*** | 0.027 |
| Pa-Ps | 0.010 | -0.017 | -0.027 | -0.001 | -0.011 |
| Pa-Ka | 0.013 | -0.006 | -0.019 | 0.011 | -0.002 |
| Ps-Ka | 0.006 | -0.002 | -0.008 | 0.023 | 0.017 |
|  |  | Total | -0.444 (p=0.94) |  | -1.09 (p=0.99) |

**S3C Table. Composition of reduced populations with eight randomly chosen individuals using** <https://www.dcode.fr/tirage-au-sort-nombre-aleatoire>. Pop: population, codes as in Table1; Ind: individual.

| **Pop** | **Ind1** | **Ind2** | **Ind3** | **Ind4** | **Ind5** | **Ind6** | **Ind7** | **Ind8** |
| --- | --- | --- | --- | --- | --- | --- | --- | --- |
| La | La03 | La04 | La05 | La06 | La07 | La08 | La09 | La10 |
| Wa | Wa01 | Wa02 | Wa03 | Wa04 | Wa06 | Wa10 | Wa11 | Wa12 |
| Mo | Mo03 | Mo04 | Mo09 | Mo10 | Mo12 | Mo13 | Mo15 | Mo16 |
| Di | Di01 | Di02 | Di03 | Di04 | Di07 | Di09 | Di11 | Di12 |
| Ki | Ki02 | Ki04 | Ki05 | Ki06 | Ki07 | Ki09 | Ki11 | Ki12 |
| Mi | Mi02 | Mi03 | Mi05 | Mi06 | Mi08 | Mi10 | Mi12 | Mi13 |
| St | St01 | St02 | St04 | St08 | St09 | St12 | St15 | St17 |
| Dw | Dw03 | Dw06 | Dw07 | Dw08 | Dw09 | Dw10 | Dw14 | Dw16 |
| Ja | Ja01 | Ja02 | Ja03 | Ja05 | Ja07 | Ja08 | Ja09 | Ja11 |
| Ds | Ds05 | Ds06 | Ds10 | Ds11 | Ds12 | Ds15 | Ds16 | Ds17 |
| Mf | Mf02 | Mf05 | Mf07 | Mf09 | Mf10 | Mf11 | Mf12 | Mf15 |
| Mt | Mt14 | Mt18 | Mt25 | Mt30 | Mt31 | Mt32 | Mt33 | Mt36 |
| Pe | Pe02 | Pe03 | Pe05 | Pe10 | Pe17 | Pe26 | Pe30 | Pe32 |
| Na | Na01 | Na04 | Na05 | Na19 | Na20 | Na28 | Na29 | Na31 |
| To | To01 | To02 | To03 | To04 | To05 | To06 | To07 | To08 |
| Nb | Nb01 | Nb07 | Nb08 | Nb09 | Nb12 | Nb13 | Nb19 | Nb23 |
| Tu | Tu02 | Tu04 | Tu05 | Tu06 | Tu08 | Tu10 | Tu12 | Tu13 |
| Sm | Sm01 | Sm02 | Sm03 | Sm04 | Sm05 | Sm06 | Sm07 | Sm08 |
| Pa | Pa01 | Pa02 | Pa05 | Pa06 | Pa07 | Pa08 | Pa09 | Pa12 |
| Ps | Ps01 | Ps04 | Ps05 | Ps06 | Ps07 | Ps08 | Ps11 | Ps12 |
| Ka | Ka02 | Ka04 | Ka05 | Ka06 | Ka07 | Ka08 | Ka09 | Ka10 |

**S3D Table. φ_ct_–values of hierarchical AMOVAs based on mtDNA (Control Region) data of *Amphiprion akallopisos* populations from the Indian Ocean** using populations composed of all available samples per population (Column 2), the first eight samples per population (Column 4), and eight randomly chosen individuals per population as mentioned in Table S3C (Column 6). Site codes as in Table 2. Significance levels: **: p < 0.01; ***: p < 0.001; ns: not significant. 95% CI: 2.5% - 97.5% Confidence interval calculated in Arlequin using 20.000 permutations.

| Groupings | Total | 95% CI | First 8 samples | 95% CI | 8 random samples | 95% CI |
| --- | --- | --- | --- | --- | --- | --- |
| (EIO)(WIO) | 0.620*** | 0.390 – 0.743 | 0.602*** | 0.389 – 0.717 | 0.610*** | 0.399 – 0.735 |
| (EIO)(Sm)(all other sites) | 0.583*** | 0.377 – 0.708 | 0.551*** | 0.359 – 0.665 | 0.560*** | 0.365 – 0.691 |
| (Sm)(all other WIO sites) | 0.341*** | 0.049 – 0.520 | 0.282*** | 0.017 – 0.473 | 0.350*** | 0.061 – 0.525 |

**S3E Table. F_ct_–values of hierarchical AMOVAs based on microsatellite data of *Amphiprion akallopisos* populations from the Indian Ocean** using populations composed of all available samples per population (Column 2), the first eight samples per population (Column 4), and eight randomly chosen individuals per population as mentioned in Table S3C (Column 6). Site codes as in Table 2. Significance levels: **: p < 0.01; ***: p < 0.001; ns: not significant. 95% CI: 2.5% - 97.5% Confidence interval calculated in Arlequin using 20.000 permutations.

| Groupings | Total | 95% CI | First 8 samples | 95% CI | 8 random samples | 95% CI |
| --- | --- | --- | --- | --- | --- | --- |
| (EIO)(WIO) | 0.110*** | 0.063 – 0.137 | 0.109*** | 0.062 – 0.143 | 0.116*** | 0.058 – 0.153 |
| (EIO)(Wa)(Tu, Nb, Sm)(all other sites) | 0.064*** | 0.035 – 0.077 | 0.066*** | 0.032 – 0.093 | 0.066*** | 0.033 – 0.093 |
| (Wa)(Tu, Nb, Sm)(all other WIO sites) | 0.013*** | 0.006 – 0.030 | 0.013** | -0.004 – 0.040 | 0.012** | -0.000 – 0.028 |
| (Tu, Nb, Sm)(all other WIO sites) | 0.014** | 0.001 – 0.027 | 0.016*** | -0.002 – 0.049 | 0.018** | 0.002 – 0.037 |
| (Wa)(all other WIO sites) | 0.013*** | 0.002 – 0.048 | 0.001 ns | -0.020 – 0.025 | -0.007 ns | -0.018 – 0.004 |
