# Supplementary material for: Sea surface currents and geographic isolation shape the genetic population structure of a coral reef fish in the Indian Ocean
Source: PLoS One. 2018 Mar 9;13(3):e0193825. doi: 10.1371/journal.pone.0193825 (PMC5844546; doi:10.1371/journal.pone.0193825)
Supplement: S1 Fig — (DOCX) [file pone.0193825.s004.docx]

[
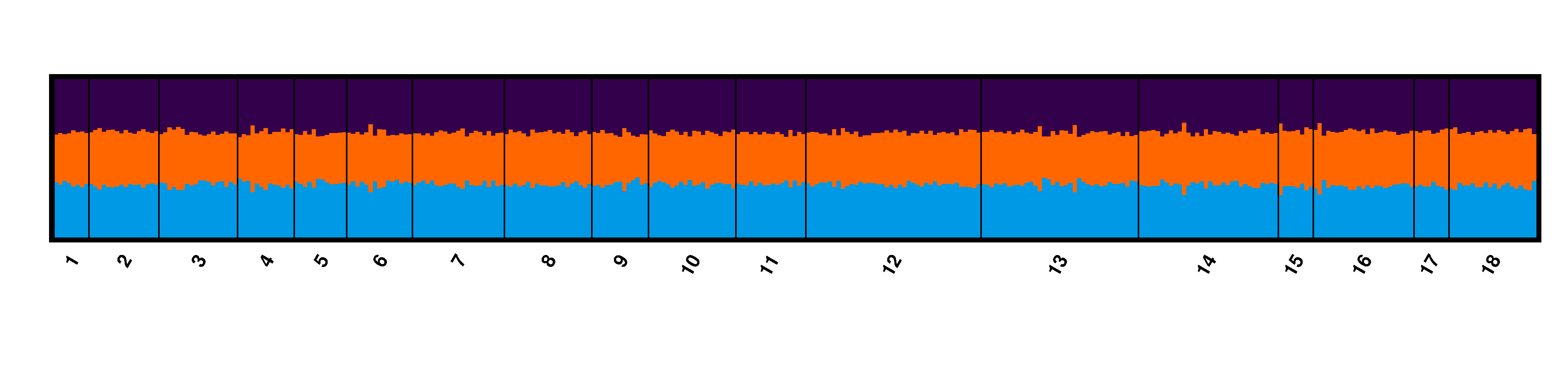
](http://clumpak.tau.ac.il/CLUMPAK_results/1484905387/K=3.MajorCluster.png)

**S1 Fig. Structure k=3 for WIO.** Colours represent different clusters. Numbers represent different populations.
