# Supplementary material for: Sea surface currents and geographic isolation shape the genetic population structure of a coral reef fish in the Indian Ocean
Source: PLoS One. 2018 Mar 9;13(3):e0193825. doi: 10.1371/journal.pone.0193825 (PMC5844546; doi:10.1371/journal.pone.0193825)
Supplement: S2 Fig — (DOCX) [file pone.0193825.s005.docx]

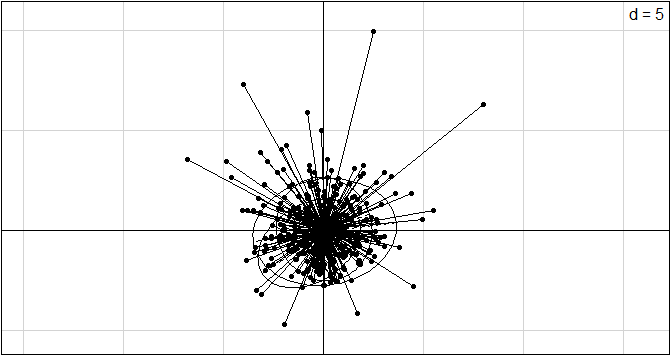


**S2 Fig. PCA of the WIO using 3 predefined groups: Northern Kenya; Madagascar; East-African coast**. Individuals indicated by dots, linked to the centre of their group, defined by 95% confidence intervals (black circles). The 95% confidence intervals of the three groups almost completely overlap.
