# Supplementary material for: Sea surface currents and geographic isolation shape the genetic population structure of a coral reef fish in the Indian Ocean
Source: PLoS One. 2018 Mar 9;13(3):e0193825. doi: 10.1371/journal.pone.0193825 (PMC5844546; doi:10.1371/journal.pone.0193825)
Supplement: S3 Fig — (DOCX) [file pone.0193825.s006.docx]

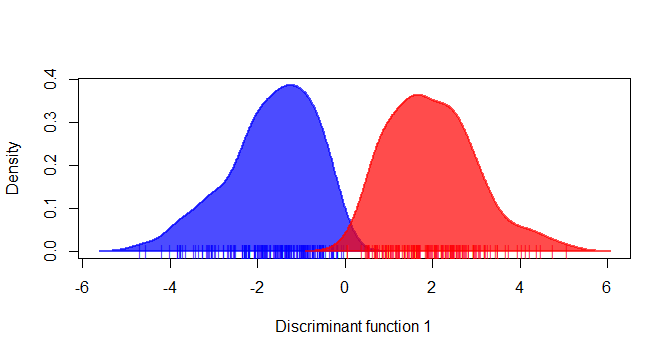


**S3 Fig. DAPC of WIO individuals defining 2 clusters**. Individuals from different populations divided among clusters as follows: Cluster 1 (blue)/ Cluster 2 (red): Di(5/8); DS(11/9); Dw(9/11); Ja(3/10); Ki(8/4); La(4/4); Mf(6/10); Mi(12/3); Mo(10/8); Mt(23/17); Na(20/12); No(8/15); Pb(26/10); Sm(5/3); St(13/8); To(4/4); Tu(6/14); Wa(11/5).
